# Fluorescein spectrofluorometric quenching detection of chloride and iodide using eight-Blue-LEDs excitation and dual solar cells

**DOI:** 10.1038/s41598-026-53458-8

**Published:** 2026-05-18

**Authors:** Ghufran K. Allawi, N. S. Turkey

**Affiliations:** 1 College of Science, Department of Chemistry, University of Baghdad, Baghdad, Iraq; 2 College of Food Science, Al-Qasim Green University, Babylon, Iraq

**Keywords:** Fluorescence quenching, Fluorescein, Chloride determination, Iodide determination, Blue LEDs excitation, Solar cell detection, Flow injection analysis, Stern-Volmer kinetics, Chemistry, Materials science, Optics and photonics, Physics

## Abstract

This study presents a novel, custom‑built fluorescence detector for the precise determination of chloride and iodide ions based on their quenching effect on fluorescein. The detection system incorporates eight blue LEDs irradiation sources arranged at 0–90° angles relative to twin solar cell detectors connected. Two fluorescence quenching strategies were developed. The injected‑mixture mode (IMFQ) exhibited linear ranges of 0.00–1.00 mM for Cl⁻ and 0.00–1.25 mM for I⁻, with detection limits of 20 µM and 10 µmol L⁻¹, respectively. The continuous‑flow mode (CFFQ) demonstrated superior sensitivity with a dispersion factor of 1.33, wider linear ranges (0.1–6.0 mM for Cl⁻ and 0.05–6.0 mM for I⁻), and lower detection limits (10 µM and 5 µM, respectively). Both strategies rely on Stern‑Volmer kinetics and were successfully applied to real samples (table salts and detergents) with excellent recoveries (95.7–108.1%).

**Supplementary Information:**

The online version contains supplementary material available at 10.1038/s41598-026-53458-8.

## Introduction

Chloride (Cl⁻) and iodide (I⁻) are chemically stable halide anions widely distributed in environmental, industrial, and biological systems. They differ significantly in their physicochemical behaviours. Chloride, characterized by a relatively small ionic radius (~ 181 pm) and low polarizability, exhibits high thermodynamic stability in aqueous media and participates predominantly in ionic interactions, coordination equilibria, and electrolyte balance without pronounced redox activity under normal conditions. In contrast, iodide possesses a substantially larger ionic radius (~ 220 pm) and markedly higher polarizability, leading to enhanced nucleophilicity and stronger spin–orbit coupling effects^1,2^. These properties influence its reactivity and interaction dynamics, particularly in photophysical and excited-state processes. Biologically, chloride is essential for osmotic and electrochemical homeostasis, whereas iodide plays a critical role as a precursor in thyroid hormone biosynthesis. Owing to their distinct electronic structures and interaction mechanisms^3^, both ions are of considerable importance in analytical chemistry, especially in fluorescence-based detection systems.

Fluorescence quenching has been established as a reliable analytical technique for halide ion determination, where the presence of chloride (Cl⁻) or iodide (I⁻) leads to a concentration-dependent decrease in fluorophore emission via collisional interactions with the excited state, following well-defined Stern–Volmer kinetics^4^. Halide-sensitive fluorescent indicators, such as quinolinium derivatives, exhibit reduced fluorescence intensity in the presence of aqueous chloride, enabling quantitative determination of Cl⁻ with measurable Stern–Volmer constants under steady-state conditions^5^. In similar systems, iodide shows even stronger quenching effects due to its larger ionic radius and higher polarizability, resulting in more efficient non-radiative deactivation of the excited fluorophore; this behaviour has been demonstrated for fluorescein-doped sensing matrices with linear Stern–Volmer responses (r² > 0.995) for I⁻ concentration ranges relevant to analytical applications^6^. Comparative studies further confirm that while all halides can contribute to quenching, the quenching efficiencies typically follow the order I⁻ > Br⁻ > Cl⁻, reflecting intrinsic differences in dynamic collisional interactions with the fluorophore^7,8^.

The sensor uses a ligand displacement mechanism: Ag⁺ quenches )2-(Furan-2-yl)-1 H-benzo[d]imidazole ‘s )FBI(( fluorescence, but Cl⁻ binds Ag⁺ more strongly, releasing FBI and restoring fluorescence. It has a detection limit of 19 µM and shows a linear calibration curve (0–60 µM) for quantification^9^. The sensor uses two fluorophores: quinine (halide-sensitive, fluorescence decreases upon halide binding) and N-(2-methacryloxyethyl) benzo[k, l]thioxanthene-3,4-dicarboximide (MBTD( (halide-insensitive, reference signal). The ratio of their fluorescence intensities (quinine/MBTD) correlates with halide concentration. The calibration curves show good linearity (R² > 0.99) with detection limits of 220 µM for Cl⁻ and 62 µM for I⁻^10^.Fluorescein and its derivatives have been extensively employed as fluorescent platforms for the detection of diverse analytes beyond halides. For instance, a fluorescein hydrazide–acetone conjugate was developed for Hg²⁺ detection, showing a turn‑on response via spirolactam ring opening with a detection limit of 7 nM^11^. Similarly, a fluorescein hydrazide probe enabled sequential detection of ClO⁻, Br⁻, and I⁻, with ClO⁻ activating the probe and then allowing Br⁻ or I⁻ to be sensed through halogenation or enhanced ring opening^12^. Another fluorescein hydrazide–benzaldehyde derivative was reported for Hg²⁺ in environmental water samples with a detection limit of 0.16 *µ*M^13^. Additionally, a simple fluorescein derivative served as a colorimetric and fluorometric sensor for Cu²⁺, with test strips for on‑site detection with a detection limit of 0.24 µM^14^.These examples underscore the structural tunability and analytical power of fluorescein‑based probes, motivating their application in the present work for halide quenching studies.

When FIA coupled with fluorescence detection, FIA enables real-time monitoring of reactions that result in either fluorescence enhancement or quenching, particularly for redox-active analytes such as ascorbic acid. Numerous fluorophores, including fluorescein, rhodamine B, and quinine sulphate, have been successfully employed as sensitive indicators in FIA platforms^15,16^. FIA has therefore become a versatile and widely applied technique in pharmaceutical analytical chemistry due its adaptability makes it particularly suitable for the determination of active pharmaceutical ingredients in complex formulations under routine analytical conditions^17–24^. Building on these principles, the present work introduces a novel fluorometric approach for the determination of ascorbic acid in pharmaceutical formulations based on fluorescence quenching under flow-injection conditions.

The novelty of this study lies in the design and integration of a custom irradiation–detection system^25^, which enables highly efficient photon collection and reproducible quenching measurements, rather than in the repetition of conventional fluorescence quenching or flow injection analysis. The device features 16 blue irradiation sources arranged in a matrix within a 100 mm brass housing. Eight of the sources are positioned at 0–180°, while the remaining eight are positioned at 0–90° in relation to twin solar-cell detectors. This configuration facilitates multi-angle excitation and enhances cumulative photon harvesting. The detectors are connected in parallel to maximize photocurrent and signal acquisition. Photons traverse a continuous‑flow quartz cell with a 2 mm irradiation path, providing uniform exposure and minimizing sample volume. This system of architecture provides several advantages over existing methodologies:

* Enhanced efficiency: Cumulative photon collection from multiple angles increases sensitivity relative to single‑beam excitation.

* Compact integration: The matrix configuration within brass housing ensures mechanical stability and reproducibility.

* Flow compatibility: The quartz flow cell enables continuous monitoring without perturbing the sample, thereby preventing secondary reactions.

* Novel detection principle: The system demonstrates that the observed quenching is purely photophysical, with no chemical reaction. This result distinguishes the present approach from previous reports that frequently assume reactive pathways.

The originality of this work is demonstrated by the unique instrumentation and experimental configuration, which have not been previously reported in the literature for chloride and iodide determination. This innovation establishes a new platform for sensitive, reproducible, and reaction‑free fluorescence quenching analysis. Under flow-injection conditions, the platform exhibits high sensitivity, reproducibility, and analytical robustness, making it suitable for precise pharmaceutical quality control based on fluorescence attenuation.

## Result and discussion

### Methodology for the classical fluorescence method

**Fig. 1:**
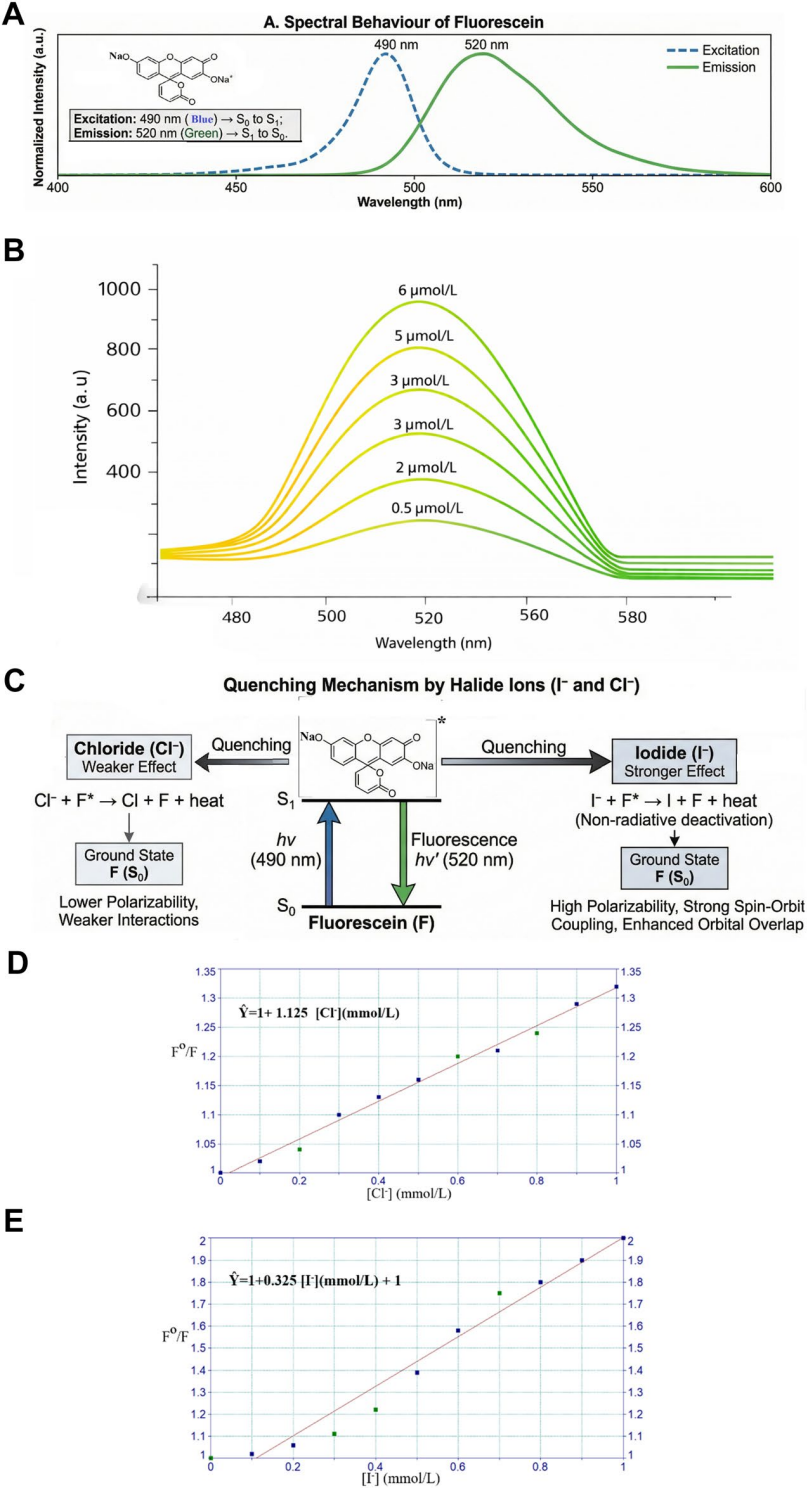
A: The excitation–emission characteristics of fluorescein define a well-separated optical window (490/520 nm) suitable for quantitative halide-induced fluorescence quenching, B:Concentration-dependent fluorescence measurements reveal a maximum response at 6.0 µM, beyond which self-quenching dominates.C: Dynamic quenching mechanism of fluorescein by halide ions. Fluorescein excited at 490 nm emits fluorescence at 520 nm. Collisional interactions with Cl⁻ or I⁻ induce non-radiative deactivation. While Fig. 2.D: Stern–Volmer analysis demonstrates linear dynamic quenching of fluorescein fluorescence by chloride ions. And Fig. 2E: Iodide exhibits enhanced Stern–Volmer quenching efficiency relative to chloride, consistent with heavy-atom–assisted nonradiative deactivation.

The spectral behaviour of fluorescein was investigated using a conventional fluorescence spectrometer (Fluorolog-3, Horiba Scientific, France) equipped with a 1 cm path-length quartz cuvette of 3 mL volume. Under these experimental conditions, the excitation spectrum exhibited a distinct maximum at 490 nm, corresponding to the absorption of blue light photons that elevate fluorescein molecules from the ground state to the first singlet excited state, while the emission spectrum was recorded at 520 nm, representing the radiative relaxation of the excited molecules back to the ground state. This excitation–emission profile is characteristic of fluorescein salts, where the xanthene chromophore provides strong absorption in the blue region and efficient fluorescence in the green region; the sharp excitation peak at 490 nm reflects efficient photon uptake, while the emission maximum at 520 nm demonstrates the stability of the excited state and its ability to yield a strong radiative signal as shown in Fig. 1A. The spectral separation between excitation and emission reduces reabsorption effects, ensuring accurate and reproducible detection, and confirms that fluorescein is highly suitable for analytical applications involving halide quenching, as excitation at 490 nm and emission at 520 nm provide a robust and sensitive fluorescence signal forming the foundation for subsequent calibration experiments. The optimal concentration of fluorescein was then investigated by preparing a series of standard solutions in the range of 0.5–6.0 µM, and as illustrated in Fig. 1B, a progressive increase in fluorescence intensity was observed with increasing fluorescein concentration, reaching a maximum response of approximately 1000 arbitrary units at 6.0 µM; further increases in concentration beyond this level resulted in a noticeable decrease in fluorescence intensity, which can be attributed to self-quenching effects arising from intermolecular interactions and energy dissipation through non-radiative thermal pathways. Accordingly, a fluorescein concentration of 6.0 µM was selected as the optimal concentration and was employed in all subsequent experiments. The quenching of fluorescein by halide ions (iodide and chloride) was subsequently examined using the same classical fluorescence spectrometer and experimental configuration, where fluorescein (6 µM) was excited at 490 nm and its emission monitored at 520 nm. A series of halide concentrations ranging from 0.1 to 1.0 mM was tested, and increasing concentrations of both iodide and chloride produced progressive quenching of the fluorescence signal, with iodide showing a stronger effect than chloride. This difference is plausibly attributed to the higher polarizability of iodide, its stronger spin–orbit coupling, and the greater probability of orbital overlap with the excited states of fluorescein, all of which enhance non-radiative deactivation, whereas chloride, with lower polarizability and weaker orbital interactions, exhibited less pronounced quenching under the same conditions as shown in Fig. 1C. The calibration plots obtained for both halides followed the Stern–Volmer relationship, confirming that the quenching mechanism is dynamic and collisional rather than static complex formation; for chloride, the Stern–Volmer plot yielded a slope of 0.325, an intercept of 0.9932, and a correlation coefficient *r* = 0.9957 as shown in Fig. 1D, while for iodide, the slope was 1.125, the intercept 0.876, and *r* = 0.9644 as shown in Fig. 1E, demonstrating strong linearity and validating that the quenching arises from collisional deactivation rather than ground-state complex formation, with iodide exhibiting markedly higher quenching efficiency compared with chloride. It attributed to iodide ion consistent with greater polarizability and enhanced spin–orbit coupling, which facilitate more efficient non-radiative deactivation pathways.

### Methodology for novel fluorescence method

**Fig. 2:**
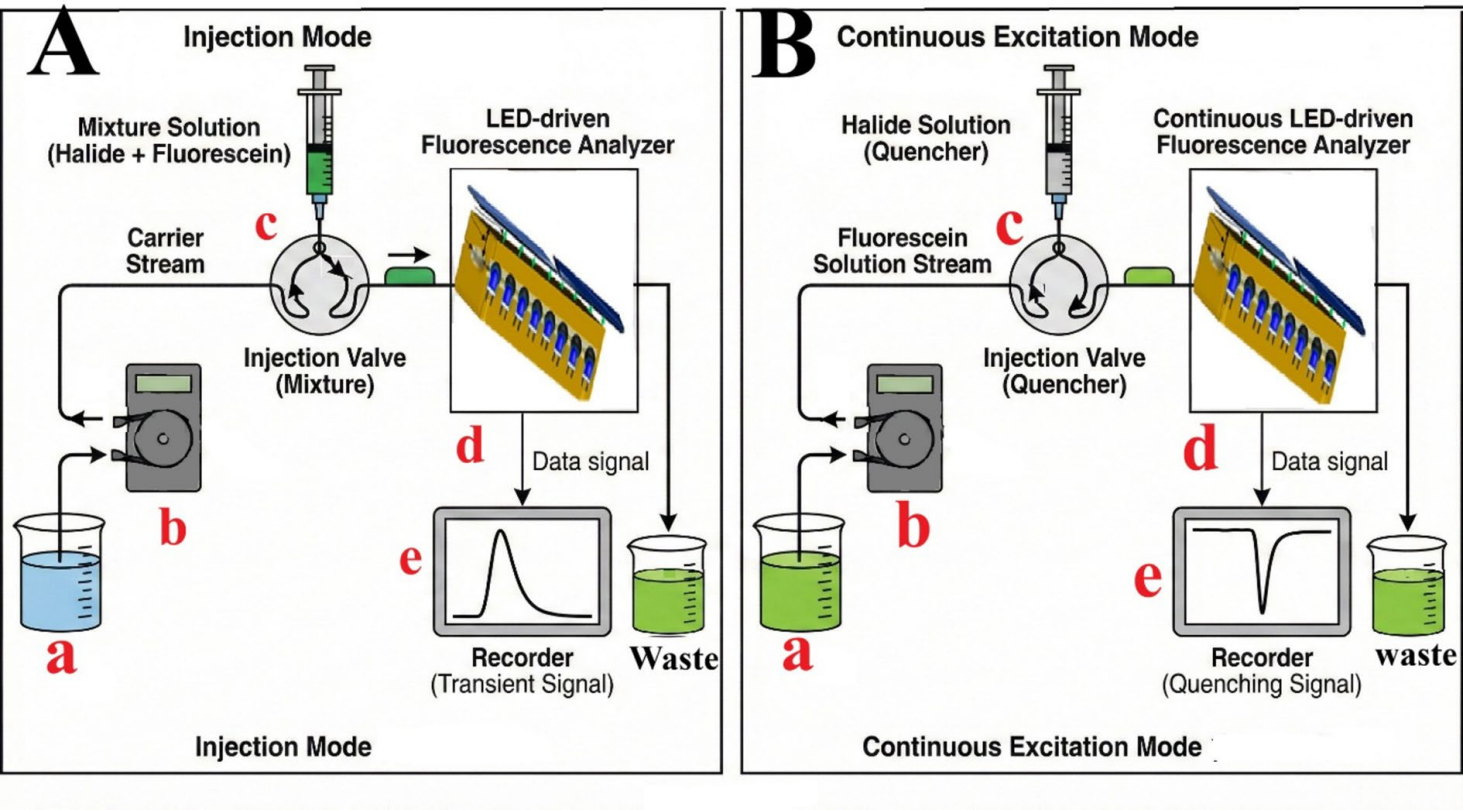
The single-line flow injection manifold used for halide determination of iodide ion and chloride via quenching of fluorescence slate. The system comprises: (**a **) Carrier or fluorescein stream, (**b**) Peristaltic pump, (**c**) Injection valve with sample loop, (**d**) Custom-built fluorescence detector (with LEDs and solar cells), and (**e**) Potentiometric recorder.

Two novel fluorescence quenching strategies were developed for the determination of chloride and iodide ions utilizing LEDs-driven excitation of fluorescein combined with solar-cell detection. The present system operates under controlled hydrodynamic conditions that regulate mass transfer and analyte–fluorophore interactions within the flow manifold as shown in Fig. 2A&B. The observed decrease in fluorescence intensity upon halide addition arises from excited-state interactions occurring during molecular collisions within the irradiated zone. Stern–Volmer analysis which confirming that the quenching proceeds predominantly via a dynamic collisional mechanism rather than ground-state complex formation as shown in Fig. 1C & D.

The first approach (Fig. 2A) is based on the Injected-Mixture Fluorescence Quenching (IMFQ) mode, where discrete aliquots of mixture solution from halide and fluorescein are introduced into a flowing carrier stream. The decrease in fluorescence intensity is monitored as a transient residual signal and compared with the reference fluorescence of the fluorescein signal.

The second approach (Fig. 2B) employs a c Continuous-Flow Fluorescence Quenching (CFFQ), in which a steady stream of fluorescein is continuously irradiated to produce a stable high-intensity baseline. The quencher is then introduced under conditions that minimise dispersion, allowing enhanced interaction between halide ions and the excited fluorophore. This configuration improves sensitivity by reducing dilution effects and maximising collisional quenching efficiency.

Both strategies rely on Stern–Volmer kinetics for quantitative evaluation while differing fundamentally in dispersion behaviour, residence time, and signal enhancement characteristics.

#### Effect of fluorescein concentration and carrier medium on fluorescence intensity

**Fig. 3:**
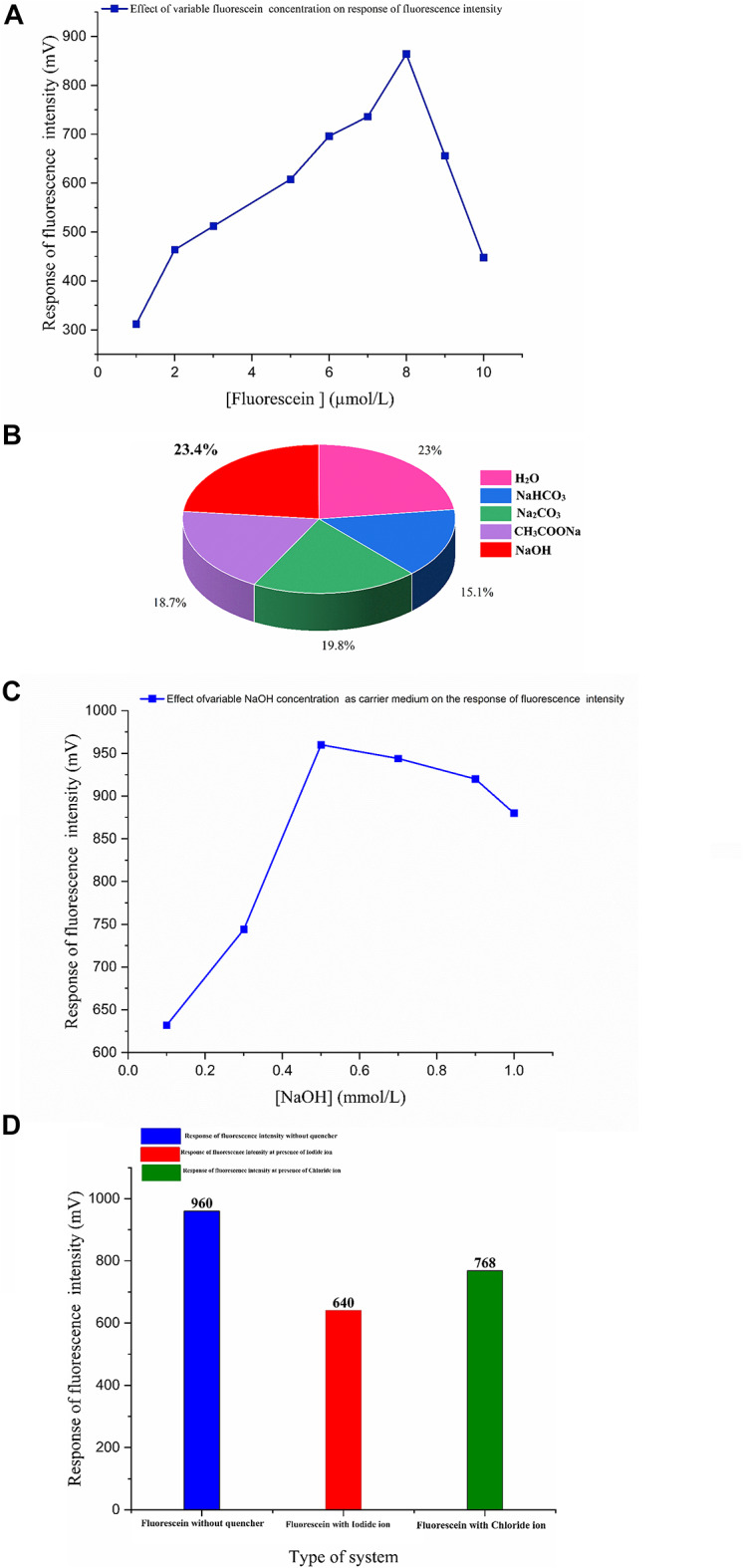
Optimization of chemical parameters for fluorescein in the flow injection system. (**A**) Effect of fluorescein concentration (1–10 µM) on fluorescence intensity, showing an optimum at 8 µM. (**B**) Effect of different carrier media (1 mmol/L) on fluorescence intensity. (**C**) Effect of sodium hydroxide concentration (0.1–1.0 mM) on fluorescence intensity, showing an optimum at 0.5 mM. (**D**) Fluorescence quenching observed for chloride and iodide under the optimized conditions (8 µM fluorescein in 0.5 mM NaOH).

The influence of fluorescein concentration on fluorescence intensity was investigated in order to optimize analytical sensitivity within the single-line flow injection system. A series of fluorescein solutions ranging from 1 to 10 µM were injected as 150 µL sample plugs into a carrier stream of deionised water. The fluorescence intensity increased progressively with concentration up to 8 µM, beyond which a decline in signal was observed as show in Fig. 3A (Table S1 (ESI)).A. This decrease is most likely attributed to self-quenching phenomena arising from molecular collisions and reabsorption of emitted photons (inner filter effect). Another plausible explanation involves aggregation of fluorescein molecules at higher concentrations, which can alter the electronic environment and reduce radiative transitions. Based on these findings, 8µM was identified as the optimal concentration for subsequent studies.

Further experiments were conducted to evaluate the effect of different carrier media on the fluorescence response of fluorescein under blue-light excitation. Sodium carbonate, sodium bicarbonate, sodium acetate, and sodium hydroxide were tested at concentrations ranging from 0.1 to 1 mM. At lower pH values, incomplete deprotonation of fluorescein hydroxyl groups reduces conjugation and fluorescence efficiency, while acidic conditions (pH < 4) promote the non‑fluorescent lactone form. The highest fluorescence intensity was obtained in alkaline medium, specifically with sodium hydroxide at 0.5 mM, as shown Fig. 3B&C. The optimal pH was approximately 10.7 (0.5 mmol L⁻¹ NaOH). This enhancement may be attributed to increased resonance stabilization of the fluorescein anion, which promotes radiative decay and facilitates a red shift toward longer emission wavelengths. A stronger contributing factor may also involve deprotonation of the fluorescein hydroxyl groups in basic conditions, leading to a more extended conjugated system and thereby amplifying fluorescence efficiency. These observations confirm that both concentration and medium composition play critical roles in maximizing the analytical performance of fluorescein for halide ion determination.

These findings clearly demonstrate that the choice of fluorescein concentration and carrier medium not only governs the overall fluorescence intensity but also dictates the sensitivity and efficiency of halide ion determination. Both chloride and iodide ions exhibited pronounced quenching effects when mixed with fluorescein at the optimized concentration of 8 µM, and the magnitude of quenching was strongly dependent on the surrounding medium. As illustrated in Fig. 3D, the optimal conditions provided a distinct enhancement in analytical performance, enabling reliable detection of both ions through their differential quenching behaviour. This confirms that the simultaneous evaluation of chloride and iodide is most effective when fluorescence excitation is carried out under carefully selected concentration and medium parameters, ensuring maximum sensitivity and repeatability of the flow injection measurements.

##### Optimization of flow rate and sample volume for enhanced LEDs-excited fluorescence

**Fig. 4:**
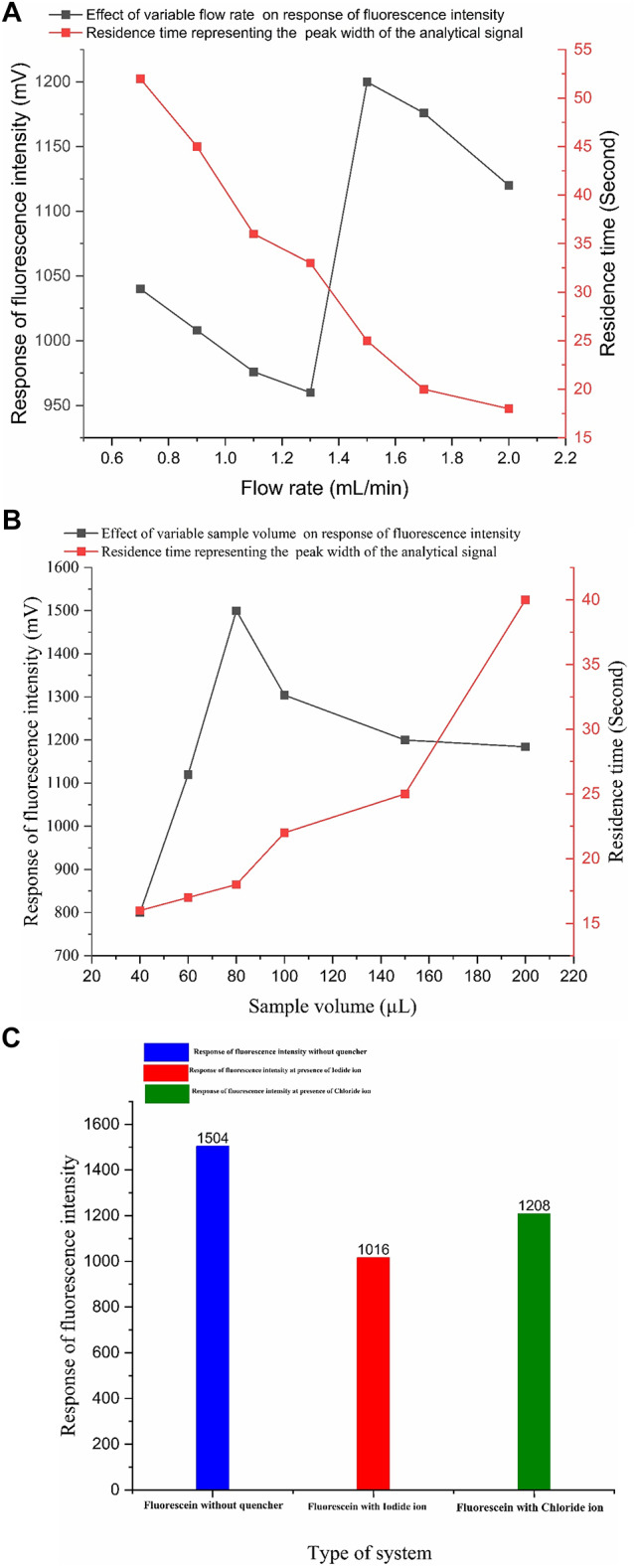
Optimization of physical parameters for the injected-mixture mode. (**A**) Effect of flow rate (0.7–2.0 mL/min) on fluorescence intensity and the residence time, with an optimum at 1.5 mL/min. (**B**) Effect of sample injection volume (40–200 µL) on fluorescence intensity and the residence time, with an optimum at 80 µL. (**C**) Fluorescence quenching signals for iodide and chloride compared to the baseline under the optimized flow conditions.

To improve the sensitivity of fluorescein fluorescence excited by blue LEDs sources for the determination of iodide and chloride ions, both the flow rate and the injected sample volume were systematically investigated under optimized chemical conditions of dye concentration and medium composition. flow rates ranging from 0.7 to 2.0 mL/min were examined (under 150 µL of sample volume). The residence time, defined as the interval between the entry of the sample zone into the flow cell (signal rise) and its complete elution back to baseline, is derived from the peak width of the analytical signal. At low flow rates, this residence time becomes prolonged, resulting in broad fluorescence responses caused by dispersion effects from diffusion and the expanded cross‑sectional area of the sample zone ahead of the detector. As the flow rate increased, sharper and more intense fluorescence signals were obtained, reaching a maximum at 1.5 mL/min. Beyond this value, the fluorescence intensity decreased, attributed to enhanced convective dispersion and dilution effects introduced by the carrier stream. Accordingly, a flow rate of 1.5 mL/min was selected as the optimum condition to achieve the highest sensitivity through maximized fluorescence response as illustrated in Fig. 4.A (Table S2(ESI).

The influence of sample volume was also evaluated using injection volumes between 40 and 200 µL optimized chemical conditions of dye concentration and medium composition and flow rate. Fluorescence intensity increased with volume up to 80 µL, reflecting the greater number of excited fluorescein molecules contributing to the signal. However, volumes exceeding 80 uL resulted in fluorescence quenching, explained by intensified molecular interactions among excited species leading to self-quenching, inner-filter effects, and partial reabsorption of emitted photons. Larger volumes additionally produced broadened responses ( high residence time) due to the expanded cross-sectional profile of the moving sample zone, which the detector integrates as cumulative photon emission as illustrated in Fig. 4.B (Table S2(ESI). Based on these findings, 80 µL. was established as the optimal injection volume to balance signal enhancement with spectral sharpness.

After fixing the physical parameters of flow rate and sample volume under halide-free conditions, the quenching behaviour of iodide and chloride was assessed. Introduction of iodide with fluorescein produced a pronounced quenching response, reducing the fluorescence intensity to 1016 mV compared with the baseline value of 1504 mV as shown in figure 4.C (Table S2(ESI)). Chloride yielded a less extensive quenching effect, with fluorescence intensity decreasing to 1208 mV under identical conditions. These results confirm that iodide exerts stronger quenching efficiency than chloride, consistent with its higher polarizability and greater capacity to promote non-radiative deactivation pathways. The optimization of flow and volume parameters thus ensured maximum sensitivity of the system, enabling reliable analytical evaluation of halide ions through LED-induced fluorescence quenching.

##### Calibration curves for chloride and iodide quenching

The fluorescence quenching of fluorescein by halide ions was quantitatively evaluated through Stern-Volmer analysis, using a reference baseline intensity of F^o^ = 1500mV in the absence of quencher.

**Fig. 5:**
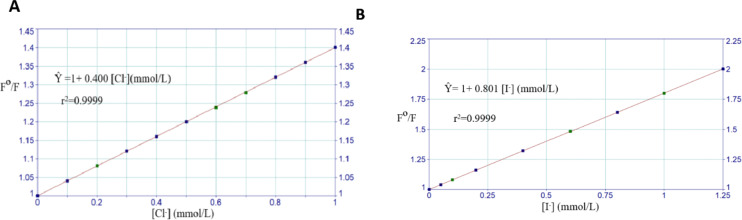
Stern-Volmer calibration plots for halide quenching in the injected-mixture mode. Linear relationship between F°/F and quencher concentration for (**A**) chloride (0.0–1.0 mM, K_sv_ = 0.399 L/mmol, *r* = 0.9999) and (**B**) iodide (0.0–1.25 mM, K_sv_ = 0.801 L/mmol, *r* = 0.9990).

Calibration curves were prepared by plotting F^º^/F against the quencher concentration [Q], where F represents the average measured fluorescence intensity in the presence of chloride or iodide. For chloride in the concentration range 0.00-1.00 mM, the Stern-Volmer relation was linear and described by the equation F^º^/F= 1 + 0.399 [Cl] mM with correlation coefficient *r* = 0. 9999 as shown un figure 5A, while for iodide in the range 0.00-1.25 mM the corresponding equation was F^º^ /F = 1 + 0.801[I^-^] mM with *r* = 0.9990 as shown in figure 5B. The calibration (table S in (ESI)) confirm that at zero halide concentration, the fluorescence intensity equals the reference value (1500 mV, F^º^ /F = 1), and that increasing halide concentration produces a continuous rase in F^º^/F with excellent linearity across the studied ranges. This linear behaviour, with intercepts at unity, demonstrates that the quenching mechanism is dynamic and collisional rather than static complex formation. The slope of the Stern-Volmer plot, K_sv_, represents the quenching constant and provides a direct measure of quenching efficiency, with chloride yielding K_sv_ = 0.399 L/mmol and iodide yielding K_sv_ = 0.801 L/mmol. When K_sv_ values fall within the moderate range (approximately 0.1-1.0 mM in the present scale), the mechanism is attributed to collisional quenching, whereas significantly higher values (greater than ~10 L/mmol) together with deviations from linearity usually indicate static quenching through ground-state complex formation. The higher value obtained for iodide indicates its stronger quenching efficiency compared with chloride, which is rationalized by the larger ionic radius, higher polarizability, and stronger spin-orbit coupling of iodide that enhance non-radiative deactivation pathways and facilitate intersystem crossing from the excited singlet to triplet states of fluorescein. In contrast, chloride, being smaller and less polarizable, interacts less effectively with the dye’s -conjugated system, resulting in a lower quenching constant. Thus, the calibration curves, supported by the tabulated data and Stern-Volmer plots, establish that both halides act through collisional quenching, with iodide exerting the more pronounced effect and providing higher analytical sensitivity within its calibration range (table S3(ESI)). Tabulated calibration results of Stern-Volmer analysis for chloride and iodide quenching of fluorescein. F= the measured fluorescence when chloride or iodide is added i.e., It shows how much fluorescence is remaining compared to the reference (F°). K_sv_ is the Stern-Volmer quenching constant, obtained as the slope of the linear plot of F^o^/F versus quencher concentration [Q]. It quantifies the efficiency of quenching. Moderate values (~0.1–1.0 L/mmol) with good linearity indicate dynamic collisional quenching, whereas very high values (>10 L /mmol.) together with deviations from linearity usually suggest static quenching through ground-state complex formation. F°/F is the Stern-Volmer ratio, defined as the baseline fluorescence intensity (F^o^) divided by the measured fluorescence intensity in the presence of quencher (F). It expresses the extent of quenching relative to the unquenched reference (i.e., F°/F = ratio of baseline fluorescence to quenched fluorescence, indicating quenching efficiency). The calibration curve is based on the Stern-Volmer equation: F^o^/F = 1 + K_sv_ [Q]. where F^o^ is the baseline fluorescence intensity in the absence of quencher, F is the fluorescence intensity in the presence of quencher, [Q]is the quencher concentration, and K_sv_ is the Stern-Volmer quenching constant.

##### Detection limits and repeatability studies for chloride and iodide

During the preparation of the calibration curves, both the detection limits and the repeatability of the measurements were evaluated. The detection limit (LOD) is defined as the lowest concentration of quencher that can be reliably distinguished from the blank signal, while repeatability refers to the consistency of replicate measurements expressed as the relative standard deviation (RSD%). For chloride, the detection limit was determined to be 0.02 mM, and replicate measurements at concentrations of 0.20 and 0.80 mM showed high repeatability with RSD% values less than 0.5%. For iodide, the detection limit was found to be 0.01 mM, and replicate measurements at 0.20 and 0.80 mM exhibited even greater precision, with RSD% values below 0.2%. These results confirm that the analytical method provides sensitive detection of both halides and excellent reproducibility across the studied concentration ranges, thereby supporting the reliability of the Stern-Volmer calibration curves.

##### Continuous fluorescence excitation for enhanced determination of halides

**Fig. 6:**
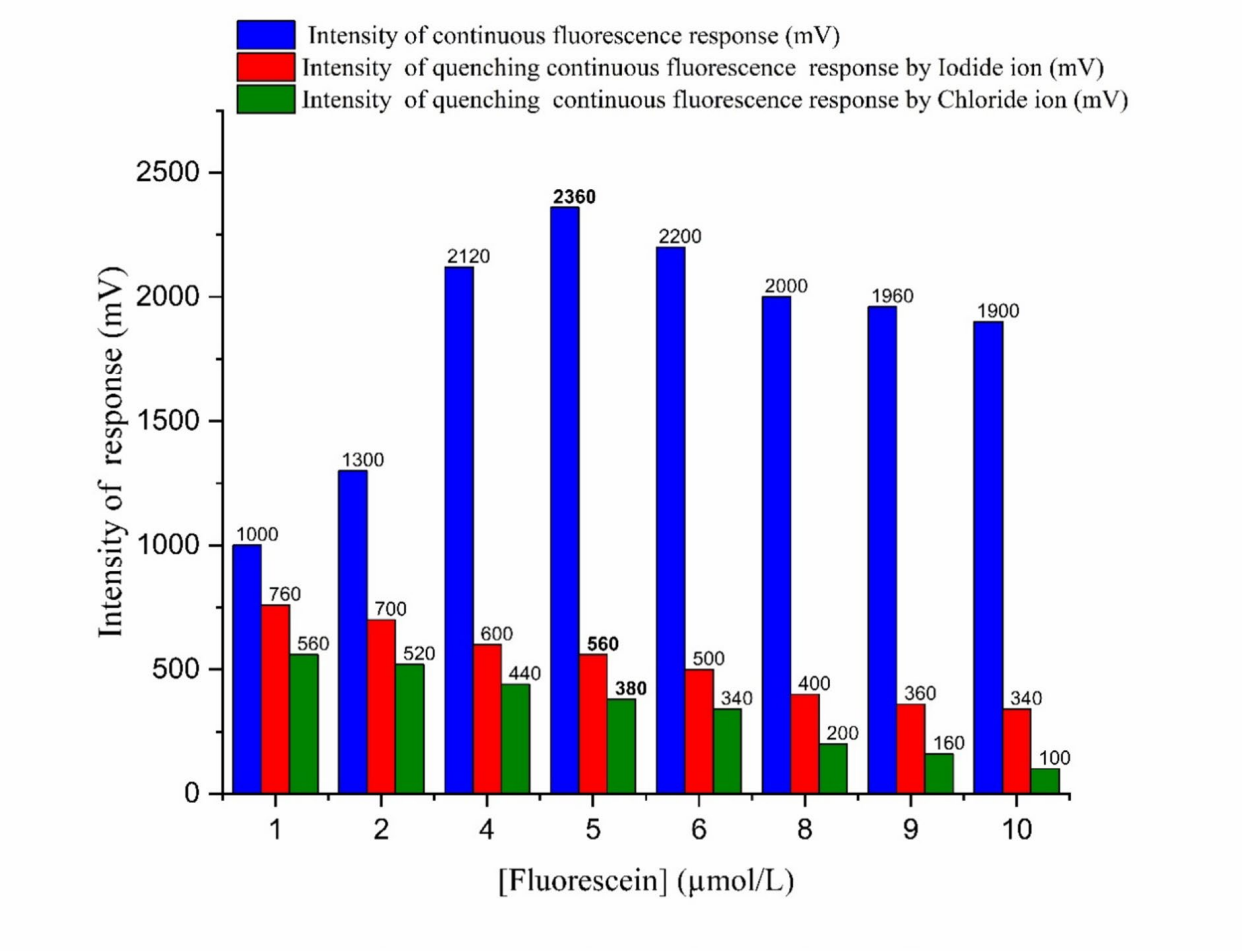
Fluorescence signal profile for the continuous fluorescence excitation mode. A stable baseline signal (2000 mV) is achieved with a continuous stream of fluorescein (8 µM in 0.5 mM NaOH), compared to the transient signal (1500 mV) obtained from the injected-mixture mode, demonstrating a dispersion factor (DF) of 1.33.

To improve the analytical determination of halide ions (iodide and chloride), a continuous fluorescence excitation system was employed using a single-line manifold through which a steady stream of fluorescein solution (8 µM ) was passed in a medium containing 0.5 mM sodium hydroxide. Under these conditions, a stable and continuous fluorescence response was obtained when the dye was excited by eight blue LEDs lamps, yielding an intensity of 2000 mV as shown in Fig. 6. This value was markedly higher than the 1500 mV intensity observed when fluorescein was introduced by discrete injection into the carrier stream, where dilution and dispersion effects around the central zone of the excited molecules reduced the effective signal. The improvement in sensitivity can be quantitatively expressed by the dispersion factor (DF), which represents the ratio of the fluorescence intensity obtained under continuous excitation to that obtained under injection mode (discrete fluorescence). Using the measured intensities I_continuous_ = 2000 mV and I_Injection_ = 1500 mV, the dispersion factor is calculated as 2000. DF =$\:\frac{{I}_{\mathrm{c}\mathrm{o}\mathrm{n}\mathrm{t}\mathrm{i}\mathrm{n}\mathrm{u}\mathrm{o}\mathrm{u}\mathrm{s}}}{{I}_{\mathrm{I}\mathrm{n}\mathrm{j}\mathrm{e}\mathrm{c}\mathrm{t}\mathrm{i}\mathrm{o}\mathrm{n}}}$ = $\:\frac{2000}{1500}$ = 1.333. This value demonstrates that continuous excitation enhances the effective fluorescence signal by approximately 33.3%, consistent with minimized dilution and dispersion compared with the injection mode, and confirming the improved analytical performance of the system. A systematic study of fluorescein concentration in the range of 1–10 µM was conducted under the fixed alkaline medium (0.5mM) of NaOH). The optimum concentration was identified as 5 µM, at which clear quenching of fluorescence and maximum sensitivity toward halide ions was achieved. At concentrations above 5 µM, the fluorescence intensity decreased due to strong self-quenching, inner-filter effects, and partial reabsorption of emitted photons. In addition, at these higher dye concentrations the quenching effect of halides became less pronounced, which is attributed to saturation of the excited states and increased probability of non-radiative decay pathways that mask the external quenching contribution. Consequently, 5 µM was established as the optimum concentration for continuous fluorescence excitation, ensuring both high sensitivity and effective halide detection (Table S4 in ESI). It is noteworthy that the optimal fluorescein concentration differs between the three configurations: 6 µM for the classical cuvette method (static, no dispersion), 8 µM for the injected mixture mode (higher dispersion, shorter interaction time), and 5 µM for the continuous flow mode (continuous excitation, reduced inner filter effects). These variations reflect the different hydrodynamic and optical conditions of each setup.

##### Influence of flow rate and sample injection volume on the fluorescence quenching of fluorescein by halide ions in a single-line flow system

**Fig. 7:**
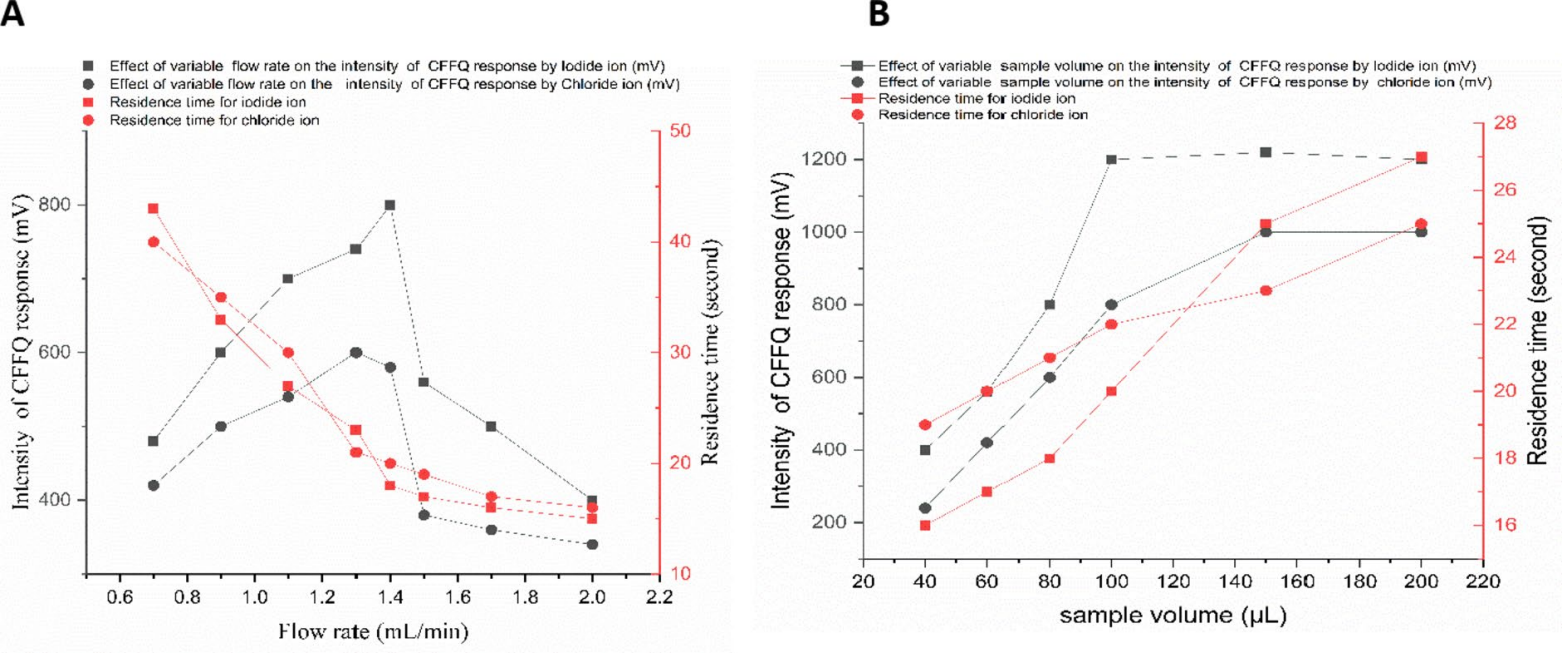
Optimization of physical parameters for the continuous fluorescence excitation mode. (A) Effect of flow rate on the quenching efficiency of 3 mM iodide and chloride and the residence time, with optima at 1.4 mL/min and 1.3 mL/min, respectively. (B) Effect of sample injection volume on quenching efficiency and the residence time, with optima at 100 µL for iodide and 150 µL for chloride.

The effect of hydrodynamic parameters on the quenching efficiency of fluorescein by chloride and iodide ions was investigated using a single-line flow injection system. A continuous stream of 5.0 µM fluorescein was maintained at variable flow rates ranging from 0.7 to 2.0 mL/min. It was observed that the quenching intensity for both halides increased proportionally with the flow rate, reaching a maximum at 1.4 mL/min for iodide (3 mM injected concentration) and 1.3 mL/min for chloride. Beyond these optimal flow rates, a discernible decline in quenching efficiency was recorded as demonstrated in Fig. 7A and (Table S.5 in ESI). The higher optimal flow rate observed for iodide (1.4 mL/min) compared to chloride (1.3 mL/min) may be attributed to the larger ionic radius and higher polarizability of the iodide ion, which facilitates more effective collisional quenching and faster mass transfer dynamics within the flowing stream even at higher rates. This attenuation in quenching at higher flow rates can be attributed to several synergistic factors. Primarily, the increased dispersion and dilution effects within the flow manifold likely reduce the effective collision frequency between the fluorophore and the quencher. Furthermore, from a kinetic perspective, higher flow rates significantly shorten the residence time of the reactants within the flow cell, providing insufficient duration for the quenching interaction or the necessary electronic transitions to occur. It is also probable that at such high rates, the limited contact time prevents efficient non-radiative deactivation pathways, such as intersystem crossing or internal/external conversion, from occurring effectively. Consequently, a flow rate of 1.4 mL/min for iodide and 1.3 mL/min for chloride was selected as the optimum operating rate for subsequent analytical investigations to ensure maximum sensitivity and repeatability quenching profiles.

The effect of sample injection volume on the quenching of fluorescein was evaluated by varying the loop size from 40 to 200 µL. The experimental data revealed a progressive increase in quenching efficiency for both halide ions as the injection volume was increased, reaching an optimum at 100 µL for iodide and 150 µL for chloride. Beyond these volumes, no significant enhancement in the quenching signal was observed, indicating a plateau in the analytical response as shown in figure 7B. The initial positive correlation between injection volume and quenching efficiency can be attributed to the reduction in the dispersion coefficient associated with larger sample plugs. As the sample volume increases, the axial dispersion at the interfaces decreases, maintaining a higher concentration of the quencher at the core of the sample zone. This minimizes the dilution effect and ensures a more concentrated interaction between the halide ions and the fluorescein stream within the detection cell. The stabilization of the signal at higher volumes suggests that a state of “steady-state” concentration has been reached, where the sample zone is sufficiently large to overcome the dispersion effects of the flow system, thus providing no further analytical gain. Consequently, injection volumes of 100 µL and 150 µL were fixed for iodide and chloride, respectively, to balance high sensitivity with minimal reagent consumption.

##### Calibration profiles and analytical validation

**Fig. 8:**
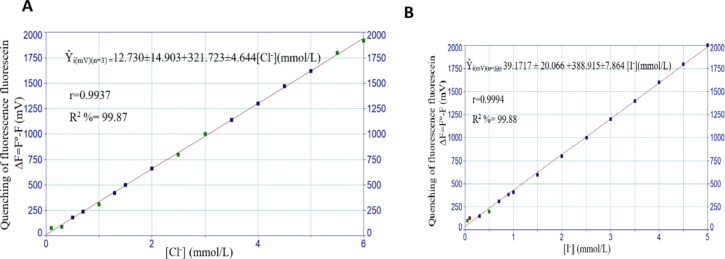
Calibration curves for halide determination using the continuous fluorescence excitation mode. Linear relationship between net quenching intensity (mV) and halide concentration for (A) chloride (0.1–6.0 mmol/L) and (B) iodide (0.05–6.0 mmol/L).

Under the optimized physical and chemical conditions, the analytical performance of the proposed flow system was established. The calibration curves exhibited a linear relationship between the halide concentration and the degree of fluorescence quenching over the range of 0.1-6 mM for chloride and 0.05-6 mM for iodide as shown in Fig. 8.A&B. The initial fluorescence intensity of fluorescein in the absence of halide ions was recorded at 2360 mV. The quenching intensity values were calculated after subtracting the blank signal from the analytical response to ensure the elimination of any background interference. The regression analysis yielded the following linear equations for chloride and iodide, respectively:

Ŷ_i(mV)(*n*=3)=_12.730 ± 14.903 + 321.723 ± 4.644 [Cl^−^] (mM) and Ŷ_i(mV)(*n*=3)=_ 39.1717 ± 20.066 + 388.915 ± 7.864 [I^−^](mM), where Ŷ represents the net quenching intensity and x denotes the concentration in mM.

At concentrations exceeding these limits, a downward deviation from linearity was observed, accompanied by a deterioration in the correlation coefficient (r).

This non-linear behaviour at higher concentrations is likely due to the saturation of the quenching sites or inner-filter effects within the detection zone. It is important to distinguish the two calibration approaches presented in this work. The Stern‑Volmer equation (F°/F = 1 + Ksv[Q]) is employed exclusively for mechanistic interpretation: the intercept at unity confirms dynamic collisional quenching, and the slope (Ksv) provides a direct measure of quenching efficiency for comparing different quenchers. For routine quantitative analysis, however, the net quenching intensity (Ŷ = F° − F, in mV) is more practical because it yields a direct linear relationship between the measured signal and analyte concentration without the need to compute a ratio. The intercept in this empirical calibration accounts for any baseline offset or blank contribution.

##### Detection limits and repeatability studies for chloride and iodide

The sensitivity of the method was further confirmed by determining the limit of detection (LOD), which was found to be 10 µM for chloride and 5 µM for iodide, indicating the suitability of the system for trace-level halide determination. The precision of the method was rigorously evaluated through repeatability studies using 1 mM and 4 mM halide concentrations for six consecutive injections (*n* = 6). The intra-day precision (repeatability within a single day) yielded a relative standard deviation percentage (RSD %) of less than 0.6%.

Furthermore, the inter-day precision (reproducibility across different days), which reflects the long-term stability of the methodology, remained below 1.0% for the same concentration levels. These results demonstrate the high reliability, robustness, and stability of the developed quenching-based flow injection manifold for routine analytical application.

#### Interference study under LEDs-solar powered spectrofluorometric system

To evaluate the selectivity of fluorescein fluorescence quenching for chloride and iodide determination, an interference study conducted using the advanced spectrofluorometric setup equipped with eight blue LEDs excitation lamps and dual solar cells as energy converters, coupled to a 100 mm transient flow cell with a 2 mm irradiation path. Under these optimized conditions, fluorescein exhibited a stable baseline intensity of 1500 m V when excited 8-blue LEDs. Potential interferents were chosen based on their frequent coexistence with halides in practical matrices: sodium hypochlorite (NaOCl), commonly present in detergent solutions where chloride is abundant, and sodium iodate (NalO_3_,), often encountered in iodized salts. Each interferent was tested at two concentration levels, 50 ppm and 100 ppm, and their effects were expressed as the percentage change in fluorescence intensity relative to the baseline. At 50 ppm, NaOCl reduced the signal by 4.2%, while NaIO_3_, caused a 5.8% decrease. At 100 ppm, the reductions increased to 7.5% and 9.6%, respectively. These values are modest compared with the pronounced quenching observed for chloride and iodide ions themselves, confirming that the primary quenching mechanism is due to direct halide-fluorescein interactions. The limited effect of these oxyanions can be attributed to their weaker orbital overlap and lower collisional efficiency with the excited states of fluorescein. Unlike halides, which induce dynamic quenching through orbital interactions and spin-orbit coupling, hypochlorite and iodate lack the electronic structure to promote efficient non-radiative decay. This interference evaluation demonstrates the analytical reliability of the LEDs solar powered spectrofluorometric system, showing that common coexisting species in detergents and salts exert only minor effects on the fluorescence response. The quenching observed for chloride and iodide is therefore confirmed to be kinetic in nature, consistent with the Stern-Volmer relationship, and not the result of static complex formation.

##### Interference study in complex solutions

In addition to sodium hypochlorite and sodium iodate, further evaluation was performed to demonstrate the efficiency of the method in complex matrices. Common ions such as bromide (Br⁻), sulfate (SO₄²⁻), and nitrate (NO₃⁻) were tested under the same optimized spectrofluorometric conditions (eight blue LEDs, dual solar cells, 100 mm flow cell, 2 mm irradiation path). Each interferent was examined at 50 ppm and 100 ppm, and the results were expressed as the percentage change in fluorescence intensity relative to the baseline Table 1.

**Table 1:** Effect of additional interferents on fluorescein quenching.

Ion (Interferent)	50 ppm (% change)	100 ppm (% change)	Observation
Br⁻	–3.5	–6.2	Weak quenching, not selective
SO₄²⁻	–2.0	–4.5	Negligible
NO₃⁻	–1.8	–4.0	Negligible

The data confirm that no significant interference was observed under the applied conditions. Bromide produced only a weak and poorly resolved quenching effect, while sulfate and nitrate showed negligible influence. This demonstrates that the proposed system maintains analytical reliability and selectivity even in complex solutions, and that the quenching observed for chloride and iodide remains specific and kinetic in nature.

#### Analytical applications and real sample analysis

To evaluate the practical applicability and “analytical prowess” of the developed methodology, the proposed system was employed for the quantitative estimation of iodide and chloride in various commercial matrices. For iodide determination, three brands of table salt were analyzed: Al-Malwiya (Baghdad, Iraq. 0.008%). Hello (Across Iraq, 0.008%), and American Garden (New York, USA, 0.006%). Regarding chloride, three detergent formulations were selected: Loyal (Jordan, 5% OCl^−^ equivalent to 3.4466 g/100 mL CI), Jif (Turkish ABS, 2.86% OCI^−^equivalent to 1.971 g/100 mL Cl), and Al-Fas (Babil, Iraq, 6% OCl^−^ equivalent to 4.136 g/100 mL CI). The analytical procedure involved the withdrawal of 0.02 ml. from 50 mM chloride sample stock and 6 mL from 0.19 mM iodide sample stock.

To circumvent complex matrix interferences, the standard addition method was implemented by spiking the sample aliquots with standard halide concentrations ranging from 0.0 to 0.5 mM (0.2, 0.3, 04, and 0.5 mM). Comparative analyses were performed using two distinct flow injection configurations: the proposed CFFQ system, where the quencher is injected into a continuous fluorophore stream, and the IMFQ mode, characterized by the injection of a pre-mixed halide-fluorophore zone into a carrier stream. The resulting responses, illustrated in Tables 2 and 3, revealed superior recovery percentages for the CFFQ mode. For chloride, the CFFQ recoveries were 99.79%, 101.89%, and 108.2%, compared to 97.69%, 100.32%, and 105.43% for the IMFQ mode. For iodide, the CFFQ system yielded recoveries of 95.69%, 98.29%, and 101.45%, while the IMFQ mode showed 94.28%, 97.25%, and 105.07%. These near-quantitative recoveries demonstrate the superior efficiency of the optimized CFFQ manifold in preserving analyte integrity and facilitating enhanced mass transfer dynamics relative to the mixture-injection approach.

Crucially, the CFFQ configuration demonstrated a significant advantage in minimizing the dispersion coefficient (D). Unlike the IMFQ mode, where the sample zone undergoes extensive axial dispersion and dilution within the carrier stream, the CFFQ system maintains the integrity of the sample plug. This minimal dispersion ensures that the quencher interacts with the fluorophore at maximum concentration, enhancing the signal-to-noise ratio and providing higher sensitivity. These near-quantitative recoveries underscore the efficiency of the optimized CFFQ manifold in preserving analyte integrity and facilitating enhanced mass transfer dynamics relative to the mixture-injection approach.

The reliability of the CFFQ method was further substantiated through rigorous statistical treatment using one-way ANOVA and paired t-tests at a 95% confidence level. The calculated t-values (t_cal_.) for all samples were determined to be lower than the critical tabulated values (t_tab_.) at the 95% confidence level, signifying the absence of detectable systematic bias and that the results of the proposed flow system are in excellent agreement with the reference IMFQ method. Furthermore, the one-way ANOVA test confirmed the homogeneity of variances across the different commercial samples (*p* > 0.05), validating that the variances between the means are statistically insignificant. All calculated t-values and F-values are less than critical values, indicating no statistically significant differences at the 95% confidence level and confirming excellent agreement between both methods and the reference values. These statistical indices confirm that the developed CFFQ manifold is a robust, highly reproducible, and accurate tool suitable for routine quality control and halide estimation in industrial and commercial formulations.

**Table 2:** Analytical applications for the determination of chloride in detergent samples and iodide in table salt samples using the proposed CFFQ and IMFQ methods, with statistical comparison.

Injected-Mixture Fluorescence Quenching (IMFQ)						
Continuous-Flow Fluorescence Quenching (CFFQ)						
Trade name	Claim value of Cl^−^ in 100 ml µ (g)	Mean of Practical Weight $\bar{\mathrm{W}}i\:(g)$±CI	Recovery %	Individual t-test	Paired t –test	ANOVA- one way
Jef OCl- 2.86% Turkey	1.971	1.977 ± 1.231	100.30	0.025 < 4.303	$\:\bar{\mathrm{W}}$_d_ = -0.070 |-3.175 | < 4.303	F_Cal_. (0.0079) < F Table (5.143)
		2.008 ± 1.028	101.89	0.130 < 4.303 < 4.303		
Loyal OCl^−^ 5% Jordan	3.447	3.367 ± 1.242	97.69	|-0.278|		
		3.440 ± 1.082	99.79	0.289 < 4.303		
AL-Fas OCl- 6% Babil, Iraq	4.136	4.361 ± 1.982	105.44	0.025 < 4.303		
		4.469± 2.982	108.05	0.1559 < 4.303		

$\:\stackrel{-}{\mathrm{W}}i\:\left(g\right)$: Mean of three replicate measurements (*n* = 3) ± CI ( confidence interval at 95% confidence level). Individual t-test comparing experimental mean with Claim value; Tableted t-value = 4.303 (*n* = 3, 95% confidence). Paired t-test comparing IMFQ and CFFQ methods for all samples; critical t-value = 4.303. One-way ANOVA F-test comparing sample groups; critical F-value = 5.143 (95% confidence).

**Table 3:** Analytical applications for the determination of iodide in table salt samples using the proposed CFFQ and IMFQ methods, with statistical comparison.

Injected-Mixture Fluorescence Quenching (IMFQ)						
Continuous-Flow Fluorescence Quenching (CFFQ)						
Trade name	Claim value of I^−^ 100 g µ (g)	Mean of Practical Weight $\:\stackrel{-}{\mathrm{W}}\:\left(g\right)$±CI	Recovery %	Individual t-test	Paired t –test ^*3^	ANOVA- one way
Al-Malwiya Potassium iodide 0.008% Baghdad-Iraq	8	7.543 ± 1.982	100.30	|-0.992| < 4.303	t_cal.=_ 0.848 < t tab = 4.303	F_cal_ = 2.274 ˂ F_tab. =_ 5.143
		7.656 ± 1.529	95.69	0.318 < 4.303		
Hello Potassium iodide 0.008% Baghdad-Iraq	8	7.780 ± 1.232	97.25	-0.767 < 4.303		
		7.863 ± 2.023	98.29	0.177 < 4.303		
American Garden Potassium iodide 0.006% New York. USA	6	6.304 ± 1.482	105.07	0.883 < 4.303		
		6.087 ± 0.238	101.452	-2.649 < 4.303		

$\:\stackrel{-}{\mathrm{W}}\:\left(g\right)$: Mean of three replicate measurements (*n* = 3) ± confidence interval at 95% confidence level. Individual t-test comparing experimental mean with reference value; critical t-value = 4.303 (*n* = 3, 95% confidence). Paired t-test comparing IMFQ and CFFQ methods for all samples; critical t-value = 4.303. One-way ANOVA F-test comparing sample groups; critical F-value = 5.143 (95% confidence).

## Materials and apparatus

### Reagent

All chemicals used were of analytical reagent grade and employed without further purification. Fluorescein sodium salt (C₂₀H₁₀Na₂O₅, M.Wt.= 376.28 g/mol, purity ≥ 98.5%) was obtained from Sigma-Aldrich (USA). Sodium hydroxide (NaOH, M.Wt = 40 g/mol, purity ≥ 98%), sodium carbonate (Na₂CO₃, M.Wt.106 g/mol, purity ≥ 99.5%), sodium bicarbonate (NaHCO₃, M.Wt **=** 105.99 g/mol, purity ≥ 99.7%), and sodium acetate (CH₃COONa, M.Wt.=82.03 g/mol ≥ 99%) were purchased from Merck (Germany). Potassium chloride (KCl, M.Wt.=74.5 g/mol ≥ 99.5%) and potassium iodide (KI = 166.00 g/mol, purity ≥ 99.5%) were supplied by BDH (UK) and Fluka (Switzerland), respectively. Detailed procedures for the preparation of all stock solutions, working standards, and real samples are provided in the Electronic Supplementary Information (ESI).

### Apparatus

**Fig. 9:**
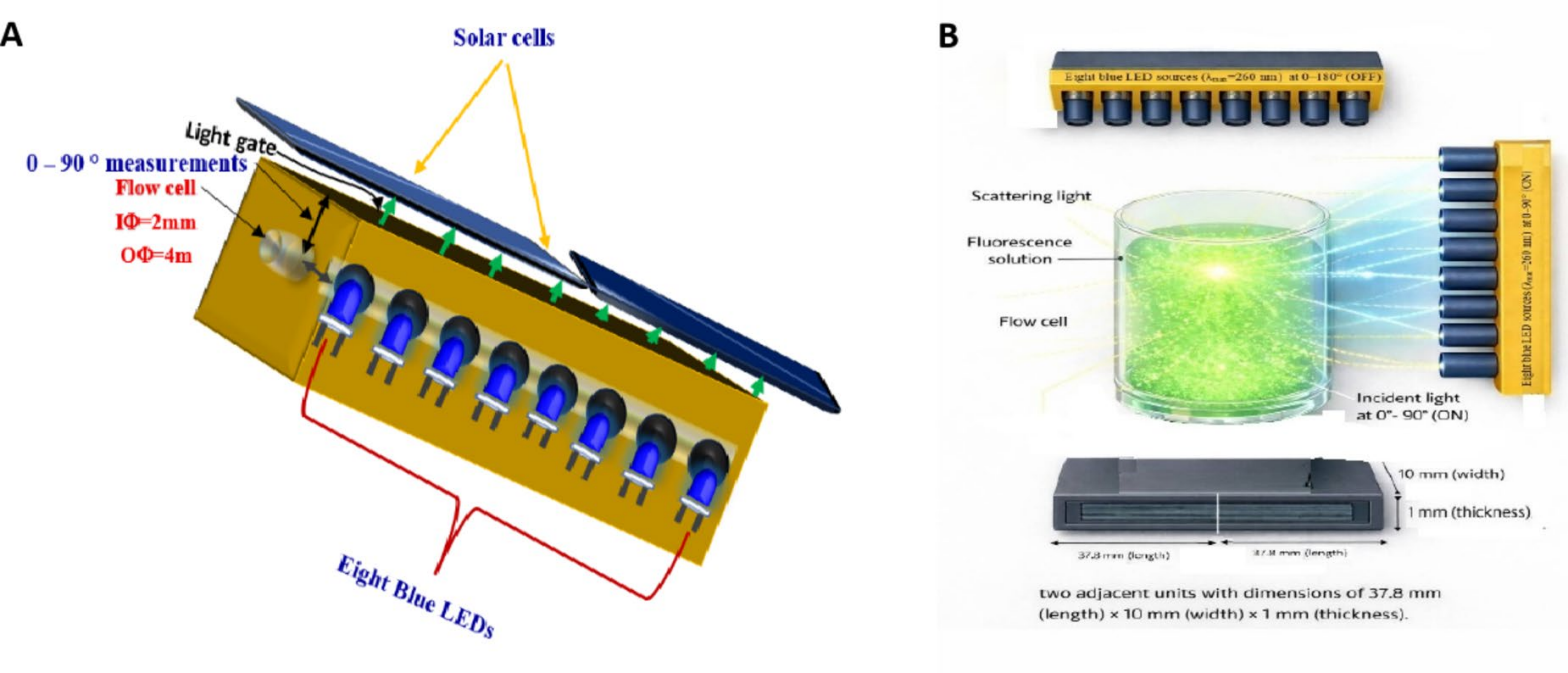
Schematic diagram of the custom-built fluorescence detector. (**A**) Top-view illustration of the brass housing showing the arrangement of eight blue LEDs excitation sources. (**B**) Fluorescence measurement of solutions using eight blue LED sources activated at 0–90° relative to the solar cell detectors, while irradiation sources at 0–180° remain switched OFF.

Dedicated to fluorescent or fluorescence‑capable solutions, where emission is detected at the 0–90° angle upon activation of the irradiation sources positioned at 0–90° relative to the solar cells. Fluorescence arises due to absorption of incident photons followed by re‑emission at longer wavelengths (Stokes shift), while quenching occurs when molecular interactions dissipate the absorbed energy non‑radiatively, reducing the fluorescence intensity. This design facilitates advanced investigation of optical interference phenomena, such as spectral symmetry and self‑absorption effects. Figure 9A illustrates the structural of custom-built instrument incorporates **sixteen blue LEDs** (5 mm diameter, λmax = 460 nm, radiant power = 1.5 W each): **eight positioned at a 0–90° angle and eight at a 0–180° angle** relative to the twin monocrystalline silicon solar cells. For fluorescence measurements in this work, **only the 0–90° set** was activated, while the 0–180° set remained off (Figure 9B). The twin solar cells (active area 37.8 × 10 mm, thickness 1 mm) were connected in parallel to maximise photocurrent. The flow manifold was constructed from PTFE tubing with an internal diameter of 2 mm and an extern.Aal diameter of 4 mm to ensure laminar flow and minimise adsorption. The flow cell was a quartz cell with an internal diameter of 2 mm, providing full geometrical compatibility with the transport tubing and ensuring a fully developed laminar flow regime.

A six-port medium-pressure injection valve (IDEX, USA) fitted with a Teflon sample loop was used for sample introduction. Signal acquisition and profiling were performed using a potentiometric chart recorder (Siemens, voltage range 1–5 V). For comparison purposes, fluorescence measurements were carried out using a commercial spectrofluorometer (FluoroMax-4, Horiba Scientific, USA) with standard 1 cm quartz cuvettes, chosen for their high optical transparency and chemical inertness, ensuring reliable fluorescence performance under optimized experimental conditions.

For comparison purposes, fluorescence measurements were carried out using a commercial spectrofluorometer (FluoroMax‑4, Horiba Scientific, USA) with standard 1 cm quartz cuvettes, chosen for their high optical transparency and chemical inertness, ensuring reliable fluorescence performance under optimized experimental conditions.

## Conclusion

A novel fluorescence-based analytical platform was successfully developed for the determination of chloride and iodide ions using fluorescein as a fluorescent probe. The proposed system integrates an eight-source blue LEDs irradiation matrix with dual solar-cell detection within a custom-designed flow-through optical cell.

The multi-angle excitation configuration significantly enhances photon harvesting and improves fluorescence signal stability compared with conventional single-beam excitation systems. Two operational modes were evaluated, namely injected-mixture fluorescence quenching (IMFQ) and continuous-flow fluorescence quenching (CFFQ), both of which demonstrated reliable Stern–Volmer behaviour over the investigated concentration ranges.

The developed platform offers several advantages, including low cost, compact instrumentation, efficient photon utilization, and compatibility with flow-injection analysis. The system provides sensitive and reproducible determination of halide ions without requiring complex optical instrumentation.

These characteristics highlight the potential of the proposed detection platform as an alternative analytical approach for fluorescence-based sensing applications.

## Electronic Supplementary Material

Below is the link to the electronic supplementary material.

Supplementary Material 1

## Data Availability

The data supporting this article have been included as part of the supplementary information (SI).
