# Supplementary material for: Fluorescein spectrofluorometric quenching detection of chloride and iodide using eight-Blue-LEDs excitation and dual solar cells
Source: Sci Rep. 2026 May 18;16:22369. doi: 10.1038/s41598-026-53458-8 (PMC13376934; doi:10.1038/s41598-026-53458-8)
Supplement: Supplementary file 1 — Supplementary Material 1 [file 41598_2026_53458_MOESM1_ESM.docx]

**Electronic *supplementary information (ESI)***

**Table S1:Fluorescence response of fluorescein under different chemical parameters**

| **Output of fluorescein a response of fluorescein under different chemical parameters** | | |
| --- | --- | --- |
| **type of parameter** | **Average output of fluorescence response Ȳ_IMF (mV) ±_ C.I. (n=3) at 95% confidence level** | **RSD %** |
| **[fluorescein] µmol/Las a sample segment (150 µL)** | | |
| **1** | **312 ± 2.037** | **0.263** |
| **2** | **464± 1.292** | **0.112** |
| **3** | **512± 2.186** | **0.172** |
| **5** | **608± 2.435** | **0.161** |
| **6** | **696± 3.279** | **0.190** |
| **7** | **736±2.534** | **0.139** |
| **8** | **864± 2.459** | **0.115** |
| **9** | **656±2.335** | **0.143** |
| **10** | **448±2.534** | **0.228** |
| **Type of carrier medium 1mmol/L** | | |
| **Deionised water** | **864 ± 2.658** | **0.124** |
| **NaHCO_3_** | **568 ±2.758** | **0.195** |
| **Na_2_CO_3_** | **744 ±2.683** | **0.145** |
| **CH_3_COONa** | **704 ±4.273** | **0.244** |
| **[NaOH] mmol/L** | | |
| **0.1** | **632 ±2.807** | **0.179** |
| **0.3** | **744 ±2.658** | **0.144** |
| **0.5** | **960 ± 2.509** | **0.105** |
| **0.7** | **944 ± 2.782** | **0.117** |
| **0.9** | **920 ±2.832** | **0.124** |
| **1** | **880 ±2.807** | **0.129** |
| **Type of system Ȳ_IMF_ or Ȳ_IMFQ (mV)_** | | |
| **Fluorescein(8µmol/L)-NaOH(0.5mmol/L) system** | **960±2.733** | **0.115** |
| **Fluorescein(8µM)-NaOH(0.5 mmol/L))-I (0.6 mmol/L) system** | **640±3.006** | **0.190** |
| **Fluorescein(8µM)-NaOH(0.5 mmol/L)-Cl (0.6 mmol/L) system** | **768±3.180** | **0.167** |

**Ȳ_IMF:_ mean of Injected-Mixture Fluorescence with out quencher  _,_ Ȳ_IMFQ:_ mean of Injected-Mixture Fluorescence Quenching in presence of quencher (residual Fluorescence). Using H_2_O as carrier stream, CI: Confidence interval t_0.05/2, 2 SD)/(√n)_, n=3 at 95% confidence level, t_0.05/2, 2_=4.303**

**Table S2:Fluorescence response of fluorescein under different physical parameters**

| Output of fluorescence a response of fluorescein under different physical parameters | | | | | | | |
| --- | --- | --- | --- | --- | --- | --- | --- |
| Type of parameter | | | | Average output of fluorescence **Ȳ_IMF_ _(mV) ± C.I. (n=3) at 95% confidence level_** | | RSD% | Residence time |
| Pump speed  (Role/min) | | Flow rate (mL/min) | | At [8] µmol/L of fluorescein as a sample segment (150 µL) and 0.5 mmol/L NaOH as carrier stream | | | |
| 5 | | 0.7 | | 1040±4.521 | | 0.435 | 52 |
| 10 | | 0.9 | | 1008±3.602 | | 0.357 | 45 |
| 15 | | 1.1 | | 976±3.279 | | 0.336 | 36 |
| 20 | | 1.3 | | 960±3.329 | | 0.347 | 33 |
| 25 | | 1.5 | | 1200±3.105 | | 0.259 | 25 |
| 30 | | 1.7 | | 1176±3.279 | | 0.279 | 20 |
| 35 | | 2 | | 1120±3.205 | | 0.286 | 18 |
| Length of sample (cm)  **(r=0.5mm)** | Sample Volume (µL), **𝒗=𝒓^𝟐^𝝅𝒉** | | At [8] µmol/L of fluorescein as a sample segment (Variable ) , [0.5] mmol/L NaOH as carrier stream and 1.5 mL/min of flow rate | | | | |
| 5.1 | 40 | | | 800±4.273 | | 0.534133 | 16 |
| 7.64 | 60 | | | 1120±4.521 | | 0.403705 | 17 |
| 10.19 | 80 | | | 1500±3.354 | | 0.22359 | 18 |
| 12.74 | 100 | | | 1304±4.025 | | 0.308637 | 22 |
| 19.11 | 150 | | | 1200±4.447 | | 0.37058 | 25 |
| 25.48 | 200 | | | 1184±4.919 | | 0.415455 | 40 |
| Type of system | | | | | Average output of fluorescein **Ȳ_IMF_ or Ȳ_IMFQ_ _(mV) ± C.I. (n=3) at 95% confidence level_** |  |  |
| Fluorescein(8µmol.L^-1^)-NaOH(0.5mmol.L^-1^) system | | | | | 1504± 3.282 | 0.218 |  |
| Fluorescein(8µM)-NaOH(0.5 mmol.L^-1^))-I (0.6 mmol.L^-1^) system | | | | | 1016±3.684 | 0.363 |  |
| Fluorescein(8µM)-NaOH(0.5 mmol.L^-1^)-Cl (0.6 mmol.L^-1^) system | | | | | 1208±4.278 | 0.354 |  |

**Ȳ_IMF:_ mean of Injected-Mixture Fluorescence with out quencher  _,_ Ȳ_IMFQ:_ mean of Injected-Mixture Fluorescence Quenching in presence of quencher (residual Fluorescence). Using H_2_O as carrier stream, Confidence interval t_0.05/2, 2 SD)/(√n)_, n=3 at 95% confidence level, t_0.05/2, 2_=4.303**

Table S3. **Calibration data for fluorescein quenching by chloride and iodide (Injected-mixture mode)**

| Type of ion | Average output of fluorescence **Ȳ_IMF_ or Ȳ_IMFQ_ _(mV) (n=3)_** | F^º^/F |
| --- | --- | --- |
| [CI^-^] (mmol/L) | Fo/F= 1 + 0.399[Cl] , (F° = 1500 mV, K_sv_ = 0.40 mmol/L,r= 0.9995) | |
| 0.00 | 1500 | 1.00 |
| 0.10 | 1441.7 | 1.041 |
| 0.20 | 1386.7 | 1.082 |
| 0.30 | 1338.7 | 1.121 |
| 0.40 | 1292.7 | 1.160 |
| 0.50 | 1250.0 | 1.200 |
| 0.60 | 1169 | 1.239 |
| 0.70 | 1172.3 | 1.279 |
| 0.80 | 1136.0 | 1.321 |
| 0.90 | 1102.7 | 1.361 |
| 1.00 | 1070.7 | 1.401 |
| [I^-^] mmol/L | Fo/F = 1 + 0.801[I^-^] mmol/L, (1500mV, K_sv_ = 0.80 mmol/L, r=0.9999) | |
| 0.00 | 1500 | 1.000 |
| 0.05 | 1442.3 | 1.040 |
| 0.10 | 1387 | 1.082 |
| 0.20 | 1292.3 | 1.082 |
| 0.40 | 1135.0 | 1.322 |
| 0.60 | 1012.7 | 1.483 |
| 0.80 | 914.0 | 1.641 |
| 1.00 | 832.7 | 1.801 |
| 1.25 | 749.3 | 2.003 |

**Ȳ_IMF:_ mean of Injected-Mixture Fluorescence with out quencher  _,_ Ȳ_IMFQ:_ mean of Injected-Mixture Fluorescence Quenching in presence of quencher (residual Fluorescence).** Reference: F° = 1500mV at zero halide; F°/F =F: Is the average of fluorescence intensity in the presence of halide ions, representing the remaining (residual) fluorescence after quenching.

**Table S4: Optimization of fluorescein concentration for continuous fluorescence mode and quenching by halide ions**

| [Fluorescein]  µmol/L | Continuous Fluorescein intensity Y_CFF_ (mV) | Quenching of continuous fluorescence by | | | |
| --- | --- | --- | --- | --- | --- |
|  |  | [I^-^]=3 mmol.L^-1^ | | [Cl^-^]=3 mmol.L^-1^ | |
|  |  | **Ȳ_CFFQ (mV) ± CI at_** **_(n=3) at 95% confidence level_** | RSD% | **Ȳ_CFFQ (mV) ± CI at_** **_(n=3) at 95% confidence level_** | RSD% |
| 1 | 1000 | 760±2.435 | 0.129 | 560±2.683 | 0.193 |
| 2 | 1300 | 700± 2.335 | 0.134 | 520±3.056 | 0.237 |
| 4 | 2120 | 600 ±2.534 | 0.170 | 440±3.280 | 0.3 |
| 5 | 2360 | 560 ±2.484 | 0.179 | 380±2.733 | 0.289 |
| 6 | 2200 | 500 ±2.807 | 0.226 | 340±2.484 | 0.294 |
| 8 | 2000 | 400 ±3.006 | 0.303 | 200±2.683 | 0.54 |
| 9 | 1960 | 360 ± 2.907 | 0.325 | 160±3.528 | 0.888 |
| 10 | 1900 | 340 ±2.708 | 0.321 | 100±3.058 | 1.23 |

**Y_CFF:_ Continuous-Flow Fluorescence with out quencher  _,_ Ȳ_IMFQ:_ mean of Continuous-Flow Fluorescence Quenching (residual Fluorescence).CI: Confidence interval t_0.05/2, 2 SD)/(√n)_, n=3 at 95% confidence level, t_0.05/2, 2_=4.303**

**Table S5: Effect of flow rate and sample volume on continuous fluorescence quenching by halide ions**

| Quenching of continuous fluorescence under different physical parameters | | | | | | | |
| --- | --- | --- | --- | --- | --- | --- | --- |
| Type of parameters | | [I^-^]=3 mmol/L | | | [Cl^-^]=3 mmol/L | | |
| Pump speed  (Role /min) | Flow rate(ml/min) | At [5] µmol/L of Fluorescein, [0.5](mmol/L) of NaOH as medium , 80 µL of sample volume and variable flow rate | | | | | |
|  |  | Ȳ_CFF (mV) ± CI at_ **_(n=3) at 95% confidence level, RSD%_** | RSD % | Residence time (second) | Ȳ_CFFQ. (mV) ± CI at_ **_(n=3) at 95% confidence level, RSD%_** | RSD % | Residence time (second) |
| 5 | 0.7 | 480 ±0.9 | 0.192 | 43 | 420±0.447 | 0.043 | 40 |
| 10 | 0.9 | 600 ± 0.83 | 0.138 | 33 | 500±1.961 | 0.158 | 35 |
| 15 | 1.1 | 700 ± 0.74 | 0.106 | 27 | 540±2.284 | 0.170 | 30 |
| 20 | 1.3 | 740 ± 0.68 | 0.092 | 23 | 600±1.912 | 0.128 | 21 |
| 23 | 1.4 | 800 ± 0.77 | 0.096 | 18 | 580±1.812 | 0.126 | 20 |
| 25 | 1.5 | 560 ± 0.59 | 0.105 | 17 | 380±2.110 | 0.224 | 19 |
| 30 | 1.7 | 500 ± 0.92 | 0.146 | 16 | 360±2.259 | 0.253 | 17 |
| 35 | 2.0 | 400 ± 0.92 | 0.23 | 15 | 340±2.408 | 0.285 | 16 |
| L.S.S (cm) (**r=0.5mm)** | V.S.S (µL)  **𝒗=𝒓^𝟐^𝝅𝒉** | At [5] µmol/L of Fluorescein, [0.5](mmol/L) of NaOH as medium and 1.3 &1.4 mL/min of flow rate for chloride ion and iodide ion and variable sample volume | | | | | |
| 5.1 | 40 | 400 ± 2.234 | 0.225 | 16 | 240±1.365 | 0.229 | 19 |
| 7.64 | 60 | 560 ± 2.085 | 0.15 | 17 | 420±1.043 | 0.1 | 20 |
| 10.19 | 80 | 800 ± 1.390 | 0.07 | 18 | 600±1.564 | 0.105 | 21 |
| 12.74 | 100 | 1200± 2.185 | 0.073 | 20 | 800±2.011 | 0.101 | 22 |
| 19.11 | 150 | 1220 ± 1.663 | 0.055 | 25 | 1000±0.968 | 0.039 | 23 |
| 25.48 | 200 | 1200 ± 2.433 | 0.082 | 27 | 1000±2.408 | 0.097 | 25 |

**L.S.S: length of sample segment, volume of sample segment . Y_CFF:_ Continuous-Flow Fluorescence with out quencher  _,_ Ȳ_IMFQ:_ mean of Continuous-Flow Fluorescence Quenching (residual Fluorescence).CI: Confidence interval t_0.05/2, 2 SD)/(√n)_, n=3 at 95% confidence level, t_0.05/2, 2_=4.303**

**Table 2. A. Analytical applications for the determination of chloride in detergent samples and iodide in table salt samples using the proposed CFFQ and IMFQ methods, with statistical comparison.**

| Injected-Mixture Fluorescence Quenching (CFFQ) | | | | | | |
| --- | --- | --- | --- | --- | --- | --- |
| Continuous-Flow Fluorescence Quenching (IMFQ) | | | | | | |
| Trade name | Claim value of Cl^-^ in 100 ml  µ (g) | Mean of Practical Weight  $\bar{W} (g)$±CI | Recovery % | Individual t-test | Paired t –test | ANOVA- one way |
| Jef  OCl-  2.86 %  Turkey | 1.971 | 1.977±1.231 | 100.30 | 0.025 < 4.303 | $\bar{W}$_d_ = -0.070  $\sigma_{n-1}^{*}$= 0.038  \|-3.175 \| < 4.303 | F_Cal.=_  0.0079  < (tab.) = 5.143 |
|  |  | 2.008±1.028 | 101.89 | 0.130 < 4.303 < 4.303 |  |  |
| Loyal  OCl^-^  5 %  Jordan | 3.447 | 3.367±1.242 | 97.69 | \|-0.278\| |  |  |
|  |  | 3.440 ±1.082 | 99.79 | 0.289 < 4.303 |  |  |
| AL-Fas  OCl-  6%  Babil ,Iraq | 4.136 | 4.361± 1.982 | 105.44 | 0.025 < 4.303 |  |  |
|  |  | 4.469± 2.982 | 108.05 | 0.1559 < 4.303 |  |  |

^*1^ $\bar{W} (g)$: Mean of three replicate measurements (n=3) ± confidence interval at 95% confidence level.
^*2^ Individual t-test comparing experimental mean with reference value; critical t-value = 4.303 (n=3, 95% confidence).
^*3^Paired t-test comparing IMFQ and CFFQ methods for all samples; critical t-value = 4.303.
^*4^ One-way ANOVA F-test comparing sample groups; critical F-value = 5.143 (95% confidence).

**Table 2. B. Analytical applications for the determination of iodide in table salt samples using the proposed CFFQ and IMFQ methods, with statistical comparison.**

| Injected-Mixture Fluorescence Quenching (CFFQ) | | | | | | |
| --- | --- | --- | --- | --- | --- | --- |
| Continuous-Flow Fluorescence Quenching (IMFQ) | | | | | | |
| Trade name | Claim value of I^-^ in 100 g  µ (g) | Mean of Practical Weight  $\bar{W} (g)$±CI | Recovery % | Individual t-test | Paired t –test | ANOVA- one way |
| Al-Malwiya  Potassium iodide  0.008 %  Baghdad-Iraq | 8 | 7.543 ± 1.982 | 100.30 | \|-0.992\| < 4.303 | t_cal.=_ 0.848 < t tab = 4.303 | F_cal_ = 2.274 ˂ F_tab. =_ 5.143 |
|  |  | 7.656 ±1.529 | 95.69 | 0.318 < 4.303 |  |  |
| Hello  Potassium iodide  0.008 %  Baghdad-Iraq | 8 | 7.780 ± 1.232 | 97.25 | -0.767 < 4.303 |  |  |
|  |  | 7.863 ± 2.023 | 98.29 | 0.177 < 4.303 |  |  |
| American Garden  Potassium iodide  0.006 %  New York. USA | 6 | 6.304 ± 1.482 | 105.07 | 0.883 < 4.303 |  |  |
|  |  | 6.087± 0.238 | 101.452 | -2.649 < 4.303 |  |  |

$\bar{W} (g)$: Mean of three replicate measurements (n=3) ± confidence interval at 95% confidence level.
 Individual t-test comparing experimental mean with reference value; critical t-value = 4.303 (n=3, 95% confidence).
Paired t-test comparing IMFQ and CFFQ methods for all samples; critical t-value = 4.303.
 One-way ANOVA F-test comparing sample groups; critical F-value = 5.143 (95% confidence).

**S.1.1:Preparation of Solutions**

Stock and Working Fluorescein Solutions: A 1.0 mmol/L fluorescein stock solution was prepared by dissolving 0.0376 g of fluorescein sodium salt in 100 mL deionized water and stored in an amber bottle at 4°C. Working fluorescein solutions (1–10 µmol/L) were freshly prepared by appropriate dilution of the stock with deionized water or with the selected carrier medium.

Carrier and Medium Solutions: A 0.1 mol/L NaOH stock solution was prepared and diluted to obtain working concentrations (0.1–1.0 mmol/L). Similarly, 0.1 mol/L stock solutions of Na₂CO₃, NaHCO₃, and CH₃COONa were prepared and diluted as required for carrier medium studies.

Halide Standard Solutions: Individual 0.1 mol/L stock solutions of chloride and iodide were prepared by dissolving 0.7455 g of KCl and 0.8300 g of KI, respectively, in 100 mL deionized water. Working calibration standards (0.05–6.0 mmol/L) were prepared by serial dilution of the stocks with deionized water.

**S.1.2.Preparation of Real Samples**

Iodized Table Salt Samples: Three commercial salt brands (Al-Malwiya, Hello, American Garden) were analyzed. Accurately weighed 10.0 g of each sample was dissolved in deionized water and diluted to 100 mL in a volumetric flask. A 6.0 mL aliquot of this solution was transferred to a 25 mL volumetric flask and diluted to volume with deionized water, yielding a nominal iodide concentration of approximately 0.19 mmol/L for analysis.

Detergent Samples: Three liquid detergent products (Loyal, Jif, Al-Fas) were analyzed. A 1.0 mL aliquot of each detergent was accurately measured, transferred to a 100 mL volumetric flask, and diluted to volume with deionized water. From this stock, 0.02 mL was taken and diluted to 25 mL with deionized water, producing a chloride concentration of approximately 50 mmol/L suitable for analysis.

Standard Addition Procedure: To overcome matrix effects, the standard addition method was applied. For each sample, five 25 mL volumetric flasks were prepared containing fixed volumes of the diluted sample solution. These were spiked with 0.0, 0.2, 0.3, 0.4, and 0.5 mmol/L of standard halide solution and diluted to volume with deionized water. Each solution was analyzed in triplicate using both CFFQ and IMFQ modes, and the halide content was determined by extrapolation of the linear standard addition plots.

**S.2.1-** Description of A custom-built photometric Analyzer

The main measuring unit of Analyzer [1] is composed of four distinct parts. As shown in Fig 1 -3.

The design of the enclosure, representing the irradiation and sensing structure of the section passing through the design:

- **The metal enclosure**: This is a piece of brass with dimensions of 100 mm (length) × 40 mm (width) × 40 mm (thickness). Brass was selected due to its ease of working with drilling tools, while copper and aluminum tend to lose structural rigidity during operation as a result of heat generated by friction, which makes them more difficult to handle. Consequently, these materials are generally avoided by Iraqi technicians.

Figure (1-3 ) shows a model of a part of the design, representing the function of the brass piece to contain the flow tube, **which has an** internal diameter of 2 mm and an external diameter of 4 mm, and the holes corresponding to the solar cells.

- **Blue irradiation sources** with a power of 1.5 W and a maximum wavelength of 460 nm: The design shows the details of the drilling diameters required to accommodate the irradiation sources, as illustrated in Figure (2) (irradiation side), and the dimensions necessary to contain the blue irradiation sources with a diameter of 5 mm. It extends to a depth of 5 mm, followed by a 7 mm diameter hole extending 10 mm to the surface. This 7 mm diameter expansion accommodates the frame and edge of the blue photodiode. The 5 mm diameter hole is followed by a 3 mm diameter hole extending 3 mm towards the center of the brass piece to touch the flow cell. This perforation is repeated eight times at angles of 0-180 degrees and 0-90 degrees relative to the detectors. Considering that the brass piece is 100 mm long, 1 cm is taken from each end, leaving a 10 mm thickness on each side to secure the brass piece to the device body. This ensures the cell remains stable during operation and prevents instability in the electronic connections.

- **Flow tube:** The 40 x 40 mm brass piece, extending 100 mm in length, is perforated at the center with a 4 mm diameter hole. This hole is necessary to accommodate the Pyrex glass flow tube, which is always 130 mm longer than the brass plate. This extension, known as the incubator approach, is 15 mm at the inlet and 15 mm at the outlet, ensuring the connection of the necessary connecting and linking pipes to stabilize the flow tube and prevent its movement in any direction.

The tube inside this hole is arranged so that the irradiation focus of the 460 nm blue light source touches the surface of the flow tube. Therefore, this design allows for the use of eight blue light-emitting diodes (1.5W), taking into account that each 10 mm of this distance accommodates only one LED source, which is immersed to a depth of 10 mm as mentioned previously. This space will be filled with a type of black silicon that prevents interference from the scattered light entering or exiting the device.

On the opposite side, the detector side, as shown in Figure 4, a hole is drilled in the surface of the brass piece, extending to 15 mm with a diameter of 9 mm. This hole is then reduced to 3 mm, extending to a depth of 3 mm (Figure 3). The total perforation length is 18 mm, which connects to the surface of the flow tube. This expansion beyond the 3 mm distance is necessary to contain as much of the generated light as possible and deliver it to the detector. These holes are repeated for the number of irradiation sources, which is 8 at the 0-180° angle, and 8 are perpendicular to the flow cell without the use of additional sensors.

Figure 3.5shows the base of the metal housing, which is the only non-perforated side. Figure 3.4 shows a longitudinal section of the 8 holes housing the light sources, corresponding to the 8 holes facing the two detectors at 0-180 degrees.

**-The detectors are twin solar cells**: two adjacent units with dimensions of 37.8 mm (length) × 10 mm (width) × 1 mm (thickness).

Based on this, the total number of blue irradiation sources is 16, divided as follows: 8 at an angle of 0-180 degrees and another 8 at 0-90 degrees relative to the solar cell detectors, with a maximum wavelength of 460 nm. These sources are immersed within a metal enclosure with holes starting from the surface with a diameter of 7 mm and extending to a depth of 10 mm, followed by a hole with a diameter of 5 mm extending to a depth of 5 mm, and then a hole with a diameter of 3 mm extending to a depth of 3 mm, for a total depth of 18 mm ( scheme1)

**Eight holes facing the two detectors, each with a diameter of 9 mm and extending to a depth of 15 mm, followed by a 3 mm diameter section extending an additional 3 mm to reach the flow cell.**


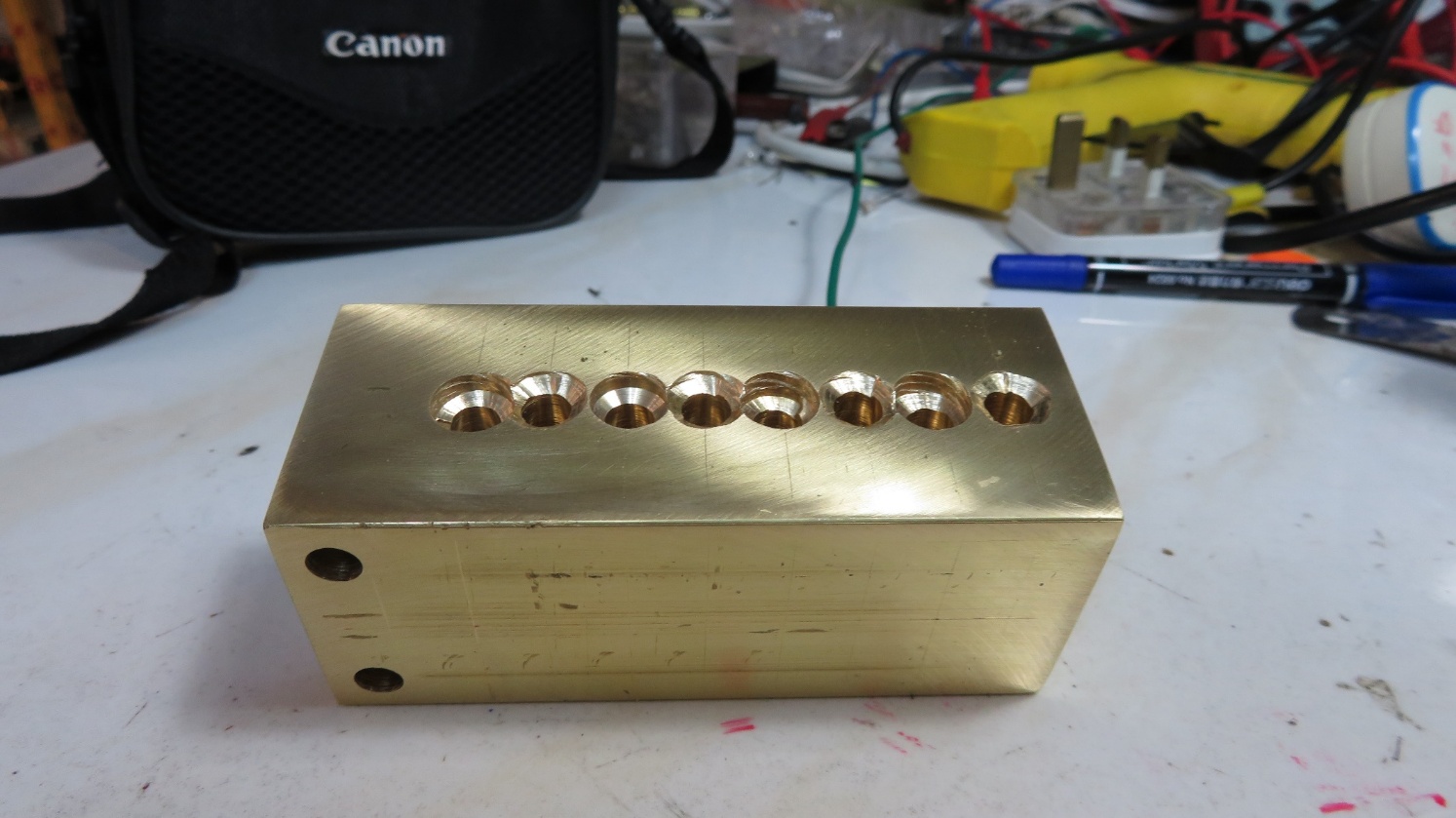


**Base of the flow cell**

**Holes for mounting the metal incubator to the base of the device**

Figure 1: Illustration of the metal incubator and the eight holes in direct alignment with the solar cells

**Locations for housing eight irradiation sources arranged at a 0–180° angle**


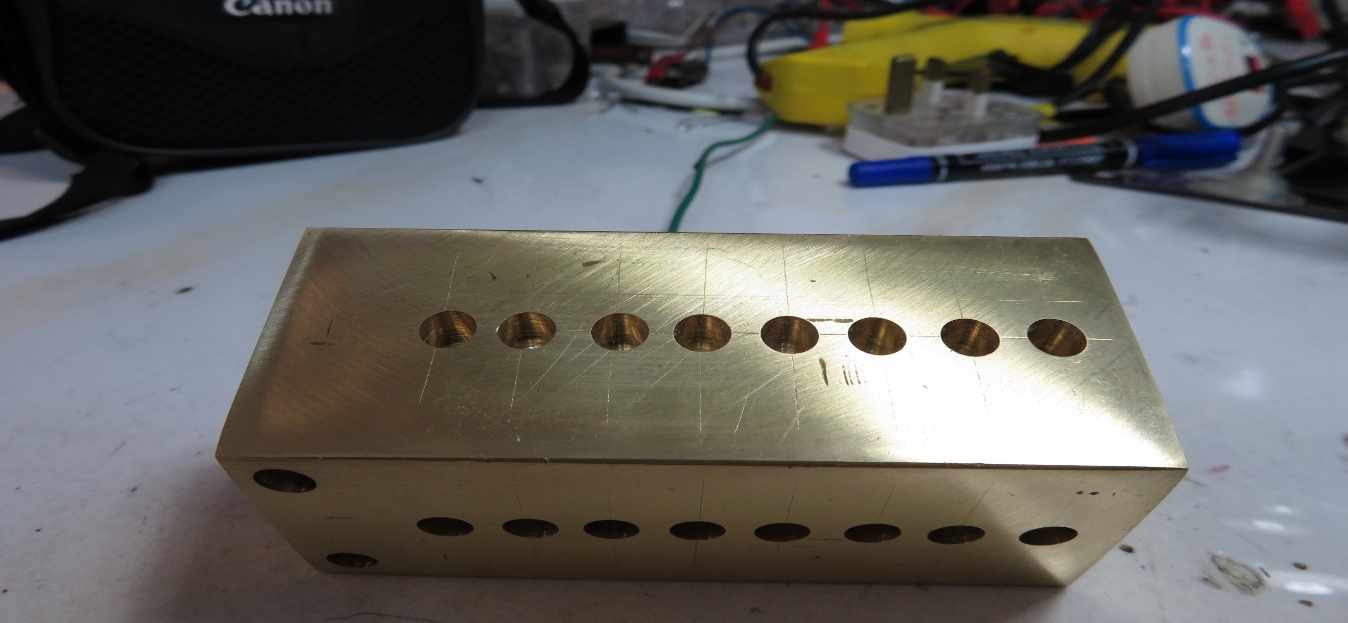


**40mm**

**40mm**

**100mm**

**Locations for housing eight irradiation sources arranged at a 0–90° angle**

Figure 2: Illustration of the metal incubator with eight holes at a 0–180° angle and another eight at a 0–90° angle relative to the two adjacent solar cells.

**Locations for housing eight irradiation sources arranged at a 0–90°relative to solar cells angle**


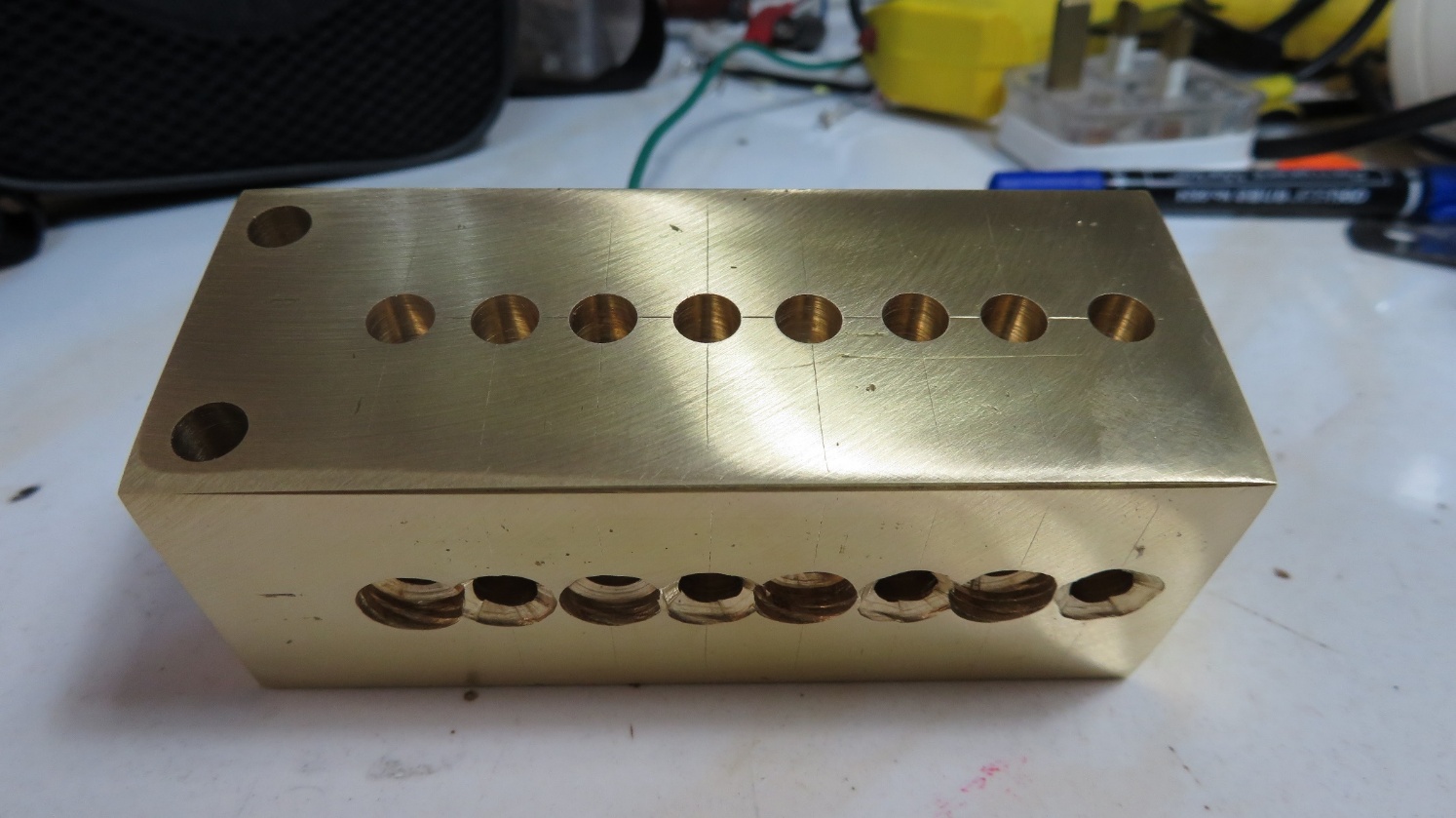


**100mm**

**40mm**

**Dual solar cells for detecting**

Figure 3: Illustration of the metal incubator and the holes (8) housing eight irradiation sources at 0–90° angles (7 mm from the surface extending to a depth of 10 mm, followed by 5 mm extending to a depth of 5 mm, and then 3 mm extending to a depth of 3 mm). In addition, the opposite holes (8) for the twin solar cells (9 mm extending to a depth of 15 mm, followed by 3 mm extending to a depth of 3 mm) are located at a 0–90° angle relative to the two detecting cells.


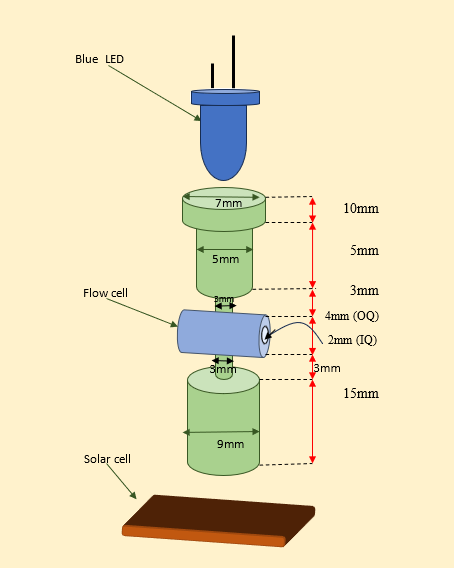
.

**Scheme 1. Longitudinal section representing NAGAYAH-IF-0-90^O^-A&T- 180^O^-2(8BLED)-2Adj-Solar cell - CFI Analyzer showing the main design features; illustrating the depths of drilling of housing of LEDs and solar cells.**

**S.2.2. Operating Manual of the A custom-built photometric Analyzer**

**Includes the following:**

1. Main switch: This is the primary power switch of the device, providing the appropriate voltage supply (220 VAC).
2. Selector switches (three in total):
   - First selector switch: Used to activate either of the operating modes. It has three positions in addition to the OFF position:

OFF - 1 - 2 - 1&2

First Position: Enables measurement of light deviation at an angle of 0–90°, covering all photon mechanics. This includes elastic photon scattering without energy loss, fluorescence formation, or deposits of any type, whether colloidal, amorphous, or crystalline.

In this mode, the nature of the formed material can be identified, determining whether the reaction produces a new amorphous phase or a modified amorphous phase compared to the applied method.

Second position: Used to select the incident light $P_{0}$and its attenuation after passing through the transient flow cell at the 0–180° position relative to the detector . This mode operates with all resulting solutions, whether colored or containing formed deposits, and the signal can be recorded independently from the 0–90° position.

Third position: Represents the combination of the signals obtained from both the 0–90° and 0–180° positions. The irradiation intensity does not necessarily have to be the same for both; the optimal intensity can be selected for either position according to the scientific and research requirements determined through the study of the optimal conditions.

According to the above, the signal outputs are three:

- The first corresponds to the 0–90° position.
- The second corresponds to the 0–180° position.
- The third corresponds to the combined signals from both positions (i.e., the sum of the two).

Accordingly, the front panel of the device comprises two switches.

1. Main power supply switch (220 VDC): The main switch supplies power at 220 VDC
2. Main selector switch is available with three options in addition to the OFF position (0–1–2–3). Electrical power is supplied from the main switch to one of these three options, depending on the selected position.
3. Supplying power to the LEDs system arranged geometrically at 0–90° with the solar cells
4. Supplying power to the LEDs system arranged geometrically at 0–180° with the solar cells

3-Supplying power simultaneously to both systems (0–90° and 0–180°), enabling mixed irradiation operation i.e., in this case, both operate together, enabling the use of mixed irradiation from the two positions as shown below ( scheme 2) :


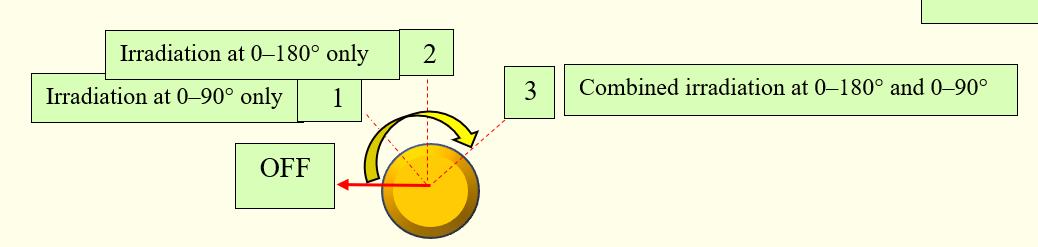


**Scheme 2: Main selector switch with three options in addition to the OFF**

**2.3.** **Principle working of the NAGAYAH‑IF‑0‑90°‑A&T‑180°‑2(8BLED)‑2Adj‑Solar Cell Analyzer**

The analyzer is a newly designed instrument that operates with three distinct measurement modes.

**First mode:** Applied to turbid solutions, where attenuation of incident blue light (λ = 460 nm) is measured at the 0–180° angle relative to the solar cell detector, as illustrated in Figure (4-A). In addition, measurement can also be performed at the 0–90° angle when the blue sources positioned at 0–90° relative to the detector are activated.


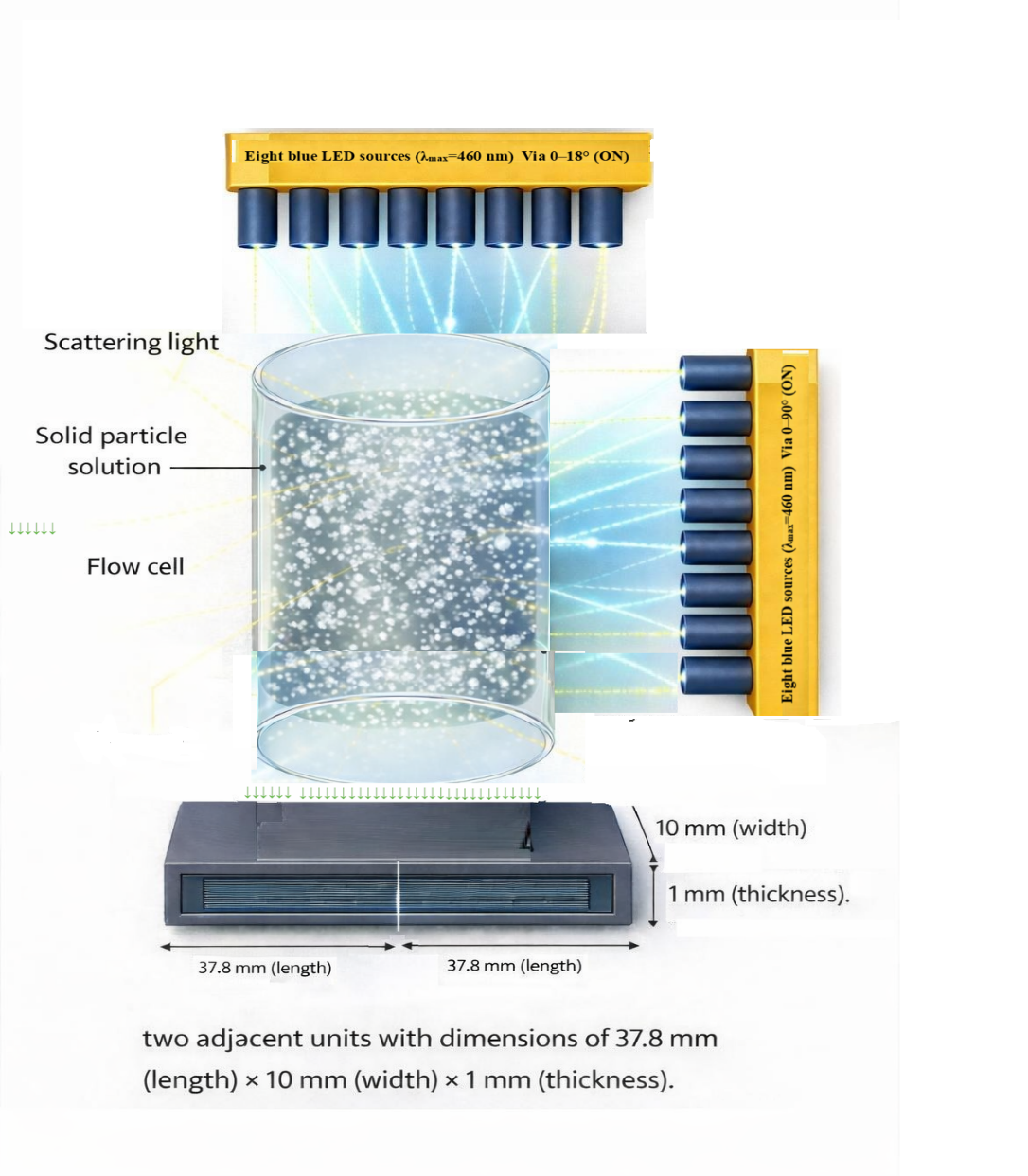

**Figure (4‑A).** **Turbidity and light scattering measurement using blue LED irradiation (λ = 460 nm): attenuation recorded at 0–180° for turbid solutions, and scattering detected at 0–90° when sources positioned at 0–90° relative to the solar cells are activated.**

**Second mode:** Used for colored solutions, in which absorbance of the irradiated beam is recorded at 0–180°, providing quantitative information on solution concentration, as shown in Figure 4-B).


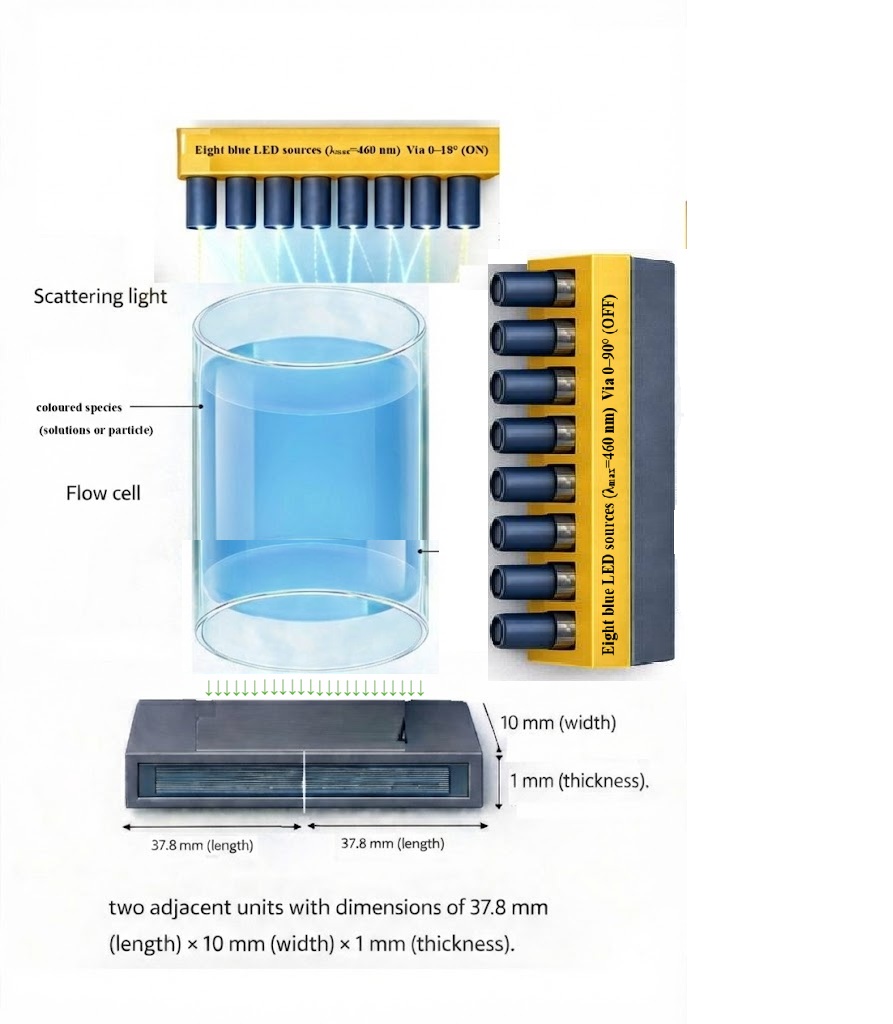


**Figure (4‑B). Attenuation of incident blue light at 0–180° relative to the solar cell detector or absorbance measurement at 0–180° for colored solutions that absorb the optimal irradiation wavelength, with 0–90° sources deactivated( switched OFF).**

**Third mode:** Dedicated to fluorescent or fluorescence‑capable solutions, where emission is detected at the 0–90° angle upon activation of the irradiation sources positioned at 0–90° relative to the solar cells. Fluorescence arises due to absorption of incident photons followed by re-emission at longer wavelengths (Stokes shift), while quenching occurs when molecular interactions dissipate the absorbed energy non‑radiatively, reducing the fluorescence intensity (Figure 3.9-C).


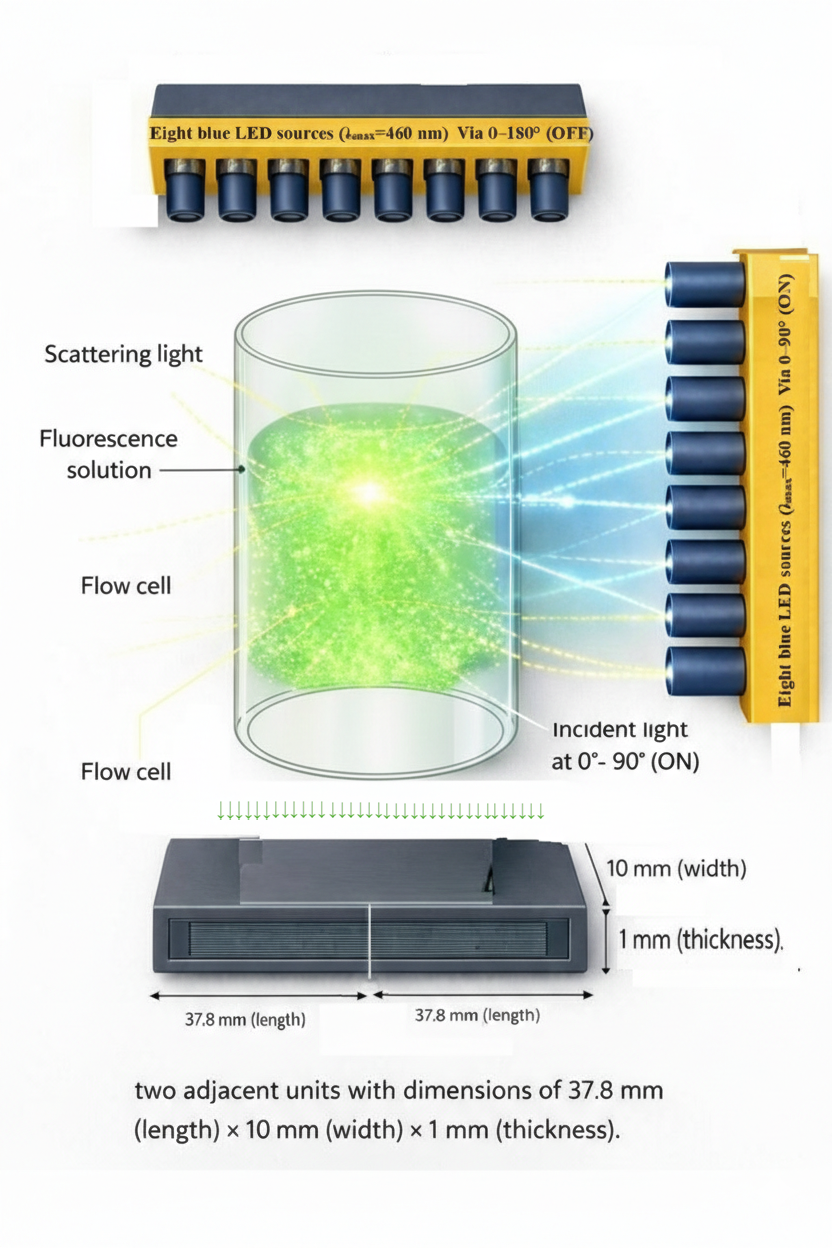


**Figure (4‑C). Fluorescence measurement of solutions using eight blue LED sources activated at 0–90° relative to the solar cell detectors, while irradiation sources at 0–180° remain switched OFF , Solar cells were used to collect fluorescence emission that is generate at 100mm distance length. All solar cells arranged to collect light at 90°.**

In all these modes, the twin solar cells act as photon-to-electric converters, ensuring that transmitted, absorbed, or emitted signals are recorded within the detection range of the cells. This design allows the analyzer to discriminate between attenuation, absorbance, and fluorescence responses, thereby enabling versatile application in chemical and pharmaceutical analysis.
